# NAD⁺ Reduction in Glutamatergic Neurons Induces Lipid Catabolism and Neuroinflammation in the Brain via SARM1

**DOI:** 10.1002/advs.202509950

**Published:** 2025-12-12

**Authors:** Zhen‐Xian Niou, Sen Yang, Andrea Enriquez, Nino A. Espinas, Anoosha Sri, Caliel D. Hines, Jason M. Tennessen, Chia‐Shan Wu, Jui‐Yen Huang, Hui‐Chen Lu

**Affiliations:** ^1^ Gill Institute for Neuroscience Indiana University Bloomington USA; ^2^ Department of Psychological and Brain Sciences Indiana University Bloomington USA; ^3^ Program in Neuroscience Indiana University Bloomington USA; ^4^ Department of Biology Indiana University Bloomington USA; ^5^ Department of Nutrition Texas A&M University College Station USA

**Keywords:** energy metabolism, inflammation, lipids, neurodegeneration, NMNAT2, SARM1

## Abstract

NAD⁺ homeostasis is vital for neuronal health, as demonstrated by the opposing roles of nicotinamide mononucleotide adenylyltransferase 2 (NMNAT2), a NAD⁺‐synthesizing enzyme, and sterile alpha and TIR motif‐containing protein 1 (SARM1), a NAD⁺ hydrolase. Neurodegenerative insults that decrease NMNAT2 activate SARM1, leading to axon loss. To understand how the NMNAT2–SARM1 axis influences brain energy metabolism, multi‐omics approaches are used to investigate the metabolic changes resulting from neuronal NMNAT2 loss. Loss of NMNAT2 in glutamatergic neurons leads to a significant metabolic shift in the cerebral cortex from glucose to lipid catabolism, reduced lipid abundance, and pronounced neurodegenerative phenotypes and motor behavioral deficits. These metabolic disturbances are accompanied by altered glial expression of enzymes regulating glucose and lipid metabolism, enhanced inflammatory signaling, and disrupted astrocytic transcriptomic profiles related to cholesterol synthesis and immune activation. Notably, SARM1 deletion in NMNAT2‐deficient mice restored lipid metabolism, astrocyte transcriptomic profiles, and mitigated neurodegeneration and motor behaviors. These findings suggest that neuronal NAD⁺ depletion triggers maladaptive, SARM1‐dependent metabolic reprogramming, shifting energy use from glucose to lipids, which in turn promotes inflammation and neurodegeneration.

## Graphical abstract

NAD⁺ homeostasis maintains neuronal integrity through opposing actions of NMNAT2 and SARM1. Loss of NMNAT2 in glutamatergic neurons reprograms cortical metabolism from glucose to lipid catabolism, depletes lipid stores, and triggers inflammation and neurodegeneration. Deletion of SARM1 restores lipid metabolism and glial homeostasis, indicating that SARM1‐dependent NAD⁺ depletion drives maladaptive metabolic remodeling in the brain.

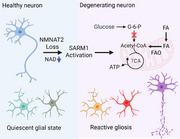

## 1 Introduction

Under normal circumstances, the brain primarily depends on glucose for energy production.^[^ ^1^, ^2^, ^3^ ^]^ As a result, deficits in glycolysis or mitochondrial pyruvate oxidation can severely impair brain function and lead to neurodegenerative conditions.^[^ ^4^, ^5^, ^6^, ^7^, ^8^ ^]^ Nicotinamide adenine dinucleotide (NAD^+^), a key cofactor that dictates the rate of glycolytic flux, serves as a vital electron acceptor and is essential for maintaining glucose‐derived ATP production. Thus, the enzymatic pathways governing NAD^+^ abundance are crucial in maintaining normal adenosine triphosphate (ATP) levels to support brain function.

Consistent with the essential role of NAD metabolism in brain health, nicotinamide mononucleotide adenylyl transferase 2 (NMNAT2) and sterile alpha and TIR motif‐containing protein 1 (SARM1), two enzymes involved in regulating steady‐state NAD^+^ levels, have been recently implicated in a wide range of neuronal disorders.^[^ ^9^, ^10^, ^11^, ^12^ ^]^ In neurons, NMNAT2 is the most abundant of the three NMNAT isoforms (NMNAT1‐3), synthesizing NAD^+^ from nicotinamide mononucleotide (NMN) via the salvage pathway.^[^ ^12^ ^]^ We and others have shown that NMNAT2 is critical to maintaining the NAD^+^/NADH redox potential and the survival of long‐range cortical axons in mice,^[^ ^13^, ^14^, ^15^ ^]^ while it is dispensable for axonal outgrowth.^[^ ^13^ ^]^ Moreover, NMNAT2 overexpression offers neuroprotection in various preclinical models of neurodegeneration and nerve injury.^[^ ^16^, ^17^, ^18^, ^19^, ^20^ ^]^ Conversely, SARM1 functions as a NAD hydrolase, and its activation leads to NAD^+^ depletion and axon degeneration.^[^ ^21^, ^22^ ^]^ SARM1 is a metabolic sensor activated when the ratio of NMN to NAD^+^ increases.^[^ ^23^ ^]^ Under normal conditions, SARM1 is kept in an inactive state because NMNAT2 converts NMN to NAD^+^ and maintains low NMN levels.^[^ ^11^, ^22^, ^24^, ^25^, ^26^, ^27^ ^]^ However, following axonal injury or under pathological conditions, NMNAT2 levels decrease, which subsequently increases NMN and activates SARM1 to trigger axon degeneration processes. Deleting SARM1 protects against the axonopathy phenotypes resulting from NMNAT2 deficiencies.^[^ ^13^, ^25^, ^28^, ^29^ ^]^

While the NMNAT2‐SARM1 axis is well‐characterized in maintaining NAD redox homeostasis and preserving axonal integrity, its broader impact on brain energy metabolism remains poorly understood. Our recent study demonstrated that neuronal NMNAT2 deficiency leads to a glycolysis deficit in distal axons, accompanied by significant neurodegenerative phenotypes.^[^ ^13^ ^]^ These findings showed that NMNAT2 loss could disrupt global brain energy metabolism and contribute to neuropathology. Substantial evidence indicates that the central nervous system (CNS) exhibits metabolic flexibility to utilize alternative fuels, such as lipids, when glucose availability is limited or glucose metabolism is disrupted.^[^ ^2^, ^30^, ^31^, ^32^, ^33^, ^34^, ^35^, ^36^, ^37^, ^38^ ^]^ We hypothesized that the disrupted NAD^+^ homeostasis caused by neuronal NMNAT2 loss exerts broader impacts by reprogramming brain energy metabolism not only in neurons but also in their neighboring cells, subsequently triggering maladaptive consequences.

In this study, we aimed to define the metabolic consequences of neuronal NMNAT2 loss in the intact brain and determine whether SARM1 deletion could rescue the metabolic aberrations resulting from NMNAT2 deficiency. To this end, we combined metabolomics approaches with proteomics and lipidomic analyses to investigate how neuronal NMNAT2 loss alters energy metabolism. We discovered that the loss of neuronal NMNAT2 leads to a significant reduction in glycolysis and an increase in lipid catabolism. Quantitative lipidomic profiling further revealed a substantial depletion of sphingolipids, glycerophospholipids, and fatty acids. This metabolic reprogramming was accompanied by robust neuroinflammation and motor behavior deficits. Complete SARM1 deletion mitigates some but not all of the metabolic alterations caused by neuronal NMNAT2 loss. Our findings suggest that neuronal NMNAT2 contributes to maintaining NAD homeostasis, and its loss is associated with a shift in brain energy metabolism from glucose toward lipid utilization.

## 2 Results

### 2.1 Loss of NMNAT2 from Glutamatergic Neurons Alters Cortical Energy Metabolism

**Figure 1:**
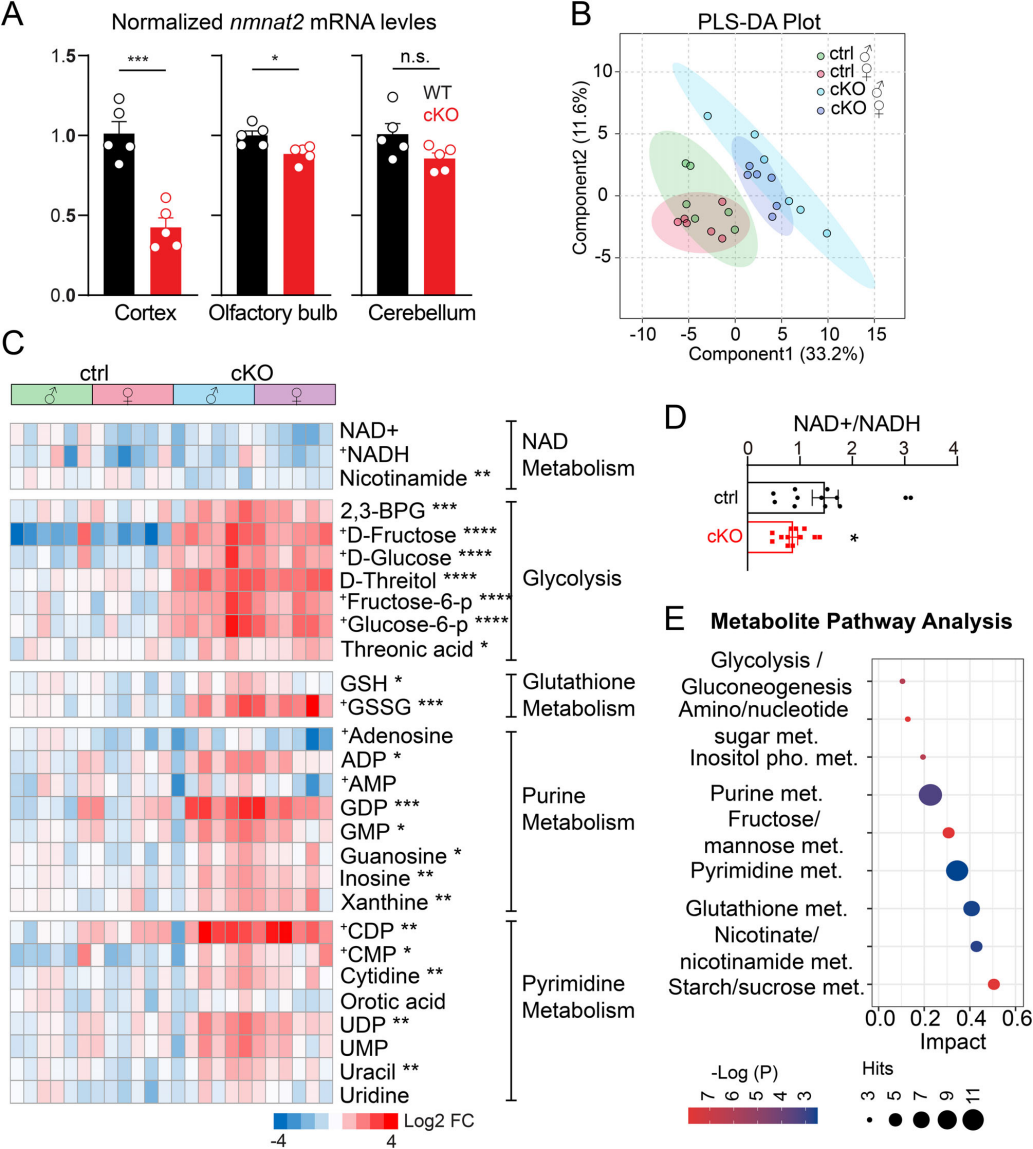
Validation of NMNAT2 loss and GC– and LC–MS metabolomic profiling reveals altered metabolic pathways in cortical glutamatergic neurons. A) Quantitative PCR analysis of *nmnat2* mRNA levels in cortex, olfactory bulbs, and cerebellum from control and cKO mice. (*n* = 5 per group; Student's *t*‐test). B) Partial least squares discriminant analysis (PLS–DA) showing dimensional reduction and separation of metabolomic profiles between individual control and cKO cortices. C) Heatmaps of log_2_‐transformed metabolite levels showing the top differentially abundant metabolites between control and cKO cortices. Metabolites are clustered by metabolic pathways, as annotated on the right. Statistical comparisons were performed on combined genders (Ctrl M+F vs. cKO M+F). (*n* = 6 per sex per group; Student's t‐test, metabolites labeled with “+” were analyzed using the Mann–Whitney test). D) Summary of NAD⁺/NADH ratios (*n* = 6 per sex per group; Mann–Whitney test). E) Bubble plot illustrates the impacts of metabolic pathways identified through Metabolite Set Enrichment Analysis (MSEA) using both upregulated and downregulated metabolites detected in cKO cortices. The *y*‐axis represents the pathway impact score, bubble size indicates the number of metabolites within each pathway, and bubble color corresponds to the ‐log(p) value. Abbreviations: cKO, cortical glutamatergic neuron‐specific deletion of NMNAT2; met., metabolism. All data are presented as mean +/‐SEM. *, *p*<0.05; **, *p*<0.01; ***, *p*<0.001; ****, *p*<0.0001. *See* *Supporting Information* *Statistics Table for detailed statistics*.

To investigate how NMNAT2 loss affects cortical energy homeostasis, we performed metabolomic profiling of P16‐P21 cortices from cortical glutamatergic neuron‐specific NMNAT2 knockout (cKO) mice and their littermate controls. The cKO mice were generated by crossing NMNAT2 conditional allele mice (NMNAT2^f/f^)^[^ ^15^ ^]^ with a Nex‐Cre transgenic mice, which expresses Cre recombinase in post‐mitotic glutamatergic neurons mainly in the cortex and hippocampus from embryonic day 11.5.^[^ ^39^ ^]^ Since these glutamatergic projection neurons constitute most of the cortical output pathways (e.g., corticospinal and corticothalamic tracts), this model allows the study of how neuronal NAD⁺ deficiency impacts axon health, brain metabolism, and neuron–glia interactions in vivo. Using quantitative PCR (qPCR), we observed that nmnat2 mRNA levels are significantly decreased in the cortex and modestly but significantly decreased in the olfactory bulb, while they remain unchanged in the cerebellum of NMNAT2 cKO mice compared to controls (**Figure** **1**A), consistent with reported Nex‐Cre activity.^[^ ^39^ ^]^

Both gas and liquid chromatography‐mass spectrometry (GC‐MS and LC‐MS) metabolomic analyses were conducted, with LC‐MS focusing on redox metabolites and nucleotides because of the known contributions of NAD^+^ to these metabolic pathways.^[^ ^40^ ^]^ Partial Least Squares Discriminant Analysis (PLS‐DA) of the metabolomic data shows distinct profiles between NMNAT2 cKO (Nex^cre/+^;NMNAT2^f/f^) and control (Nex^cre/+^;NMNAT2^f/+^) cortices, independent of sex (Figure 1B). Significant decreases in nicotinamide (Figure 1C) and NAD/NADH ratios in cKO cortices (Figure 1D) underscore the critical role of NMNAT2 in maintaining cortical NAD^+^ homeostasis. Notably, cKO cortices contain significantly higher levels of sugar derivatives, oxidized glutathione, and purine (adenine and guanine) and pyrimidine (uracil, thymine, and cytosine) nucleotides compared to controls (Figure 1C). Metabolite Set Enrichment Analysis (MSEA), conducted using MetaboAnalyst 4.0,^[^ ^41^ ^]^ further indicated significant alterations in several pathways: glycolysis, amino acid metabolism, nucleotide sugar metabolism, purine/pyrimidine metabolism, glutathione metabolism, and starch/sugar metabolism (Figure 1E). These metabolic changes suggest that the loss of neuronal NMNAT2 results in marked alterations in several metabolic pathways.

### 2.2 NMNAT2 Loss in Glutamatergic Neurons Disrupted Glucose and Lipid Metabolism

**Figure 2:**
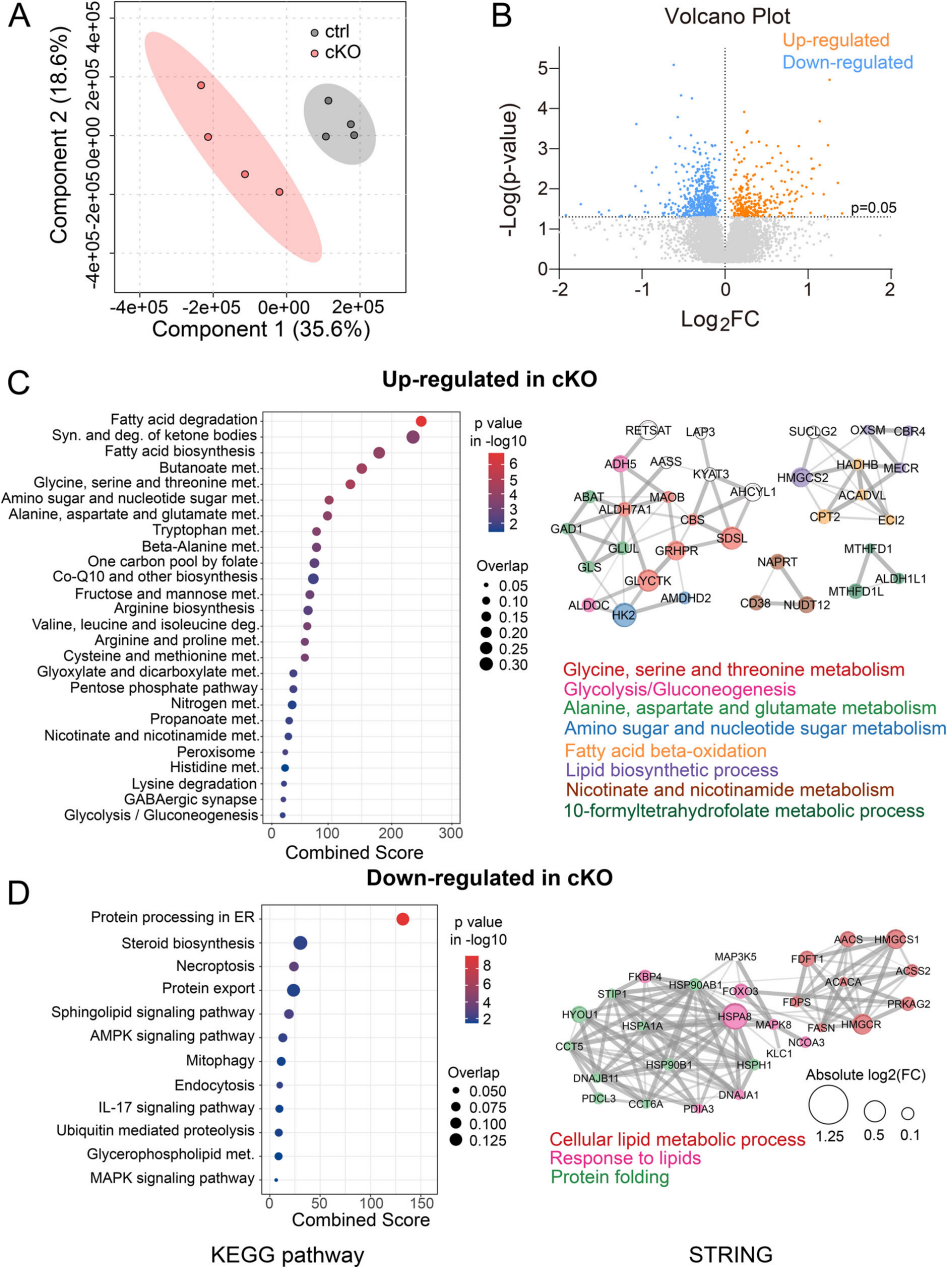
Proteomic profiling of NMNAT2 cKO and control cortices. A) PLS‐DA plot shows distinct proteomic profiles of control and cKO cortices. B) Volcano plot displaying differentially expressed proteins in cKO cortices relative to controls, highlighting both upregulated and downregulated proteins based on fold‐change and significance thresholds. C,D) Summary graphs for KEGG pathway enrichment analysis (left panels) and STRING protein–protein interaction analysis (right panels) with upregulated C) and downregulated proteins D) in cKO cortices. In the bubble plots, bubble size indicates the overlap ratio of proteins associated with each pathway, and the x‐axis represents the combined enrichment score derived from Enrichr analysis. The STRING networks display key hub proteins, where edge thickness represents interaction confidence and node size reflects relative fold change. Sample size: *n* = 4 males per group.

To gain further insights into the metabolic shifts and determine whether the protein abundance of key enzymes in the identified metabolic pathways could explain the metabolic changes caused by NMNAT2 loss, we conducted proteomic profiling of P16‐P21 control and NMNAT2 cKO cortices. PLS‐DA analysis of the proteomic data revealed distinct proteomic profiles between the control and cKO groups (**Figure** **2**A). In NMNAT2 cKO cortices, 292 proteins were significantly upregulated, while 504 proteins were significantly downregulated compared to the control (Figure 2B). KEGG pathway enrichment analyses were conducted separately with the significantly upregulated and downregulated proteins to identify overrepresented pathways altered in NMNAT2 cKO cortices (Figure 2C,D left panels). Notably, the fatty acid β‐oxidation (FAO) pathway emerged as the most significantly upregulated pathway. In contrast, pathways related to protein processing and lipid biosynthetic pathways, such as steroid biosynthesis, were found to be downregulated. STRING protein–protein interaction network cluster analysis, used to identify key hub proteins, revealed that many upregulated proteins were involved in amino acid and sugar metabolism networks. Interestingly, the analysis also highlighted several upregulated proteins associated with FAO and lipid biosynthetic processes (Figure 2C right panel). For the downregulated proteins in NMNAT2 cKO cortices, STRING identified several proteins within networks related to lipid metabolism, response to lipids, and protein folding (Figure 2D right panel). These findings suggest that NMNAT2 loss triggers the upregulation of catabolic pathways while suppressing anabolic (biosynthetic) pathways involved in lipid metabolism. The observed proteomic changes in NMNAT2 cKO cortices emphasize profound alterations in fatty acid/lipid metabolism and protein homeostasis following the loss of NMNAT2 in glutamatergic neurons.

### 2.3 Neuronal NMNAT2 Loss Leads to a Metabolic Shift from Glycolysis Toward FAO and Ketogenesis

**Figure 3:**
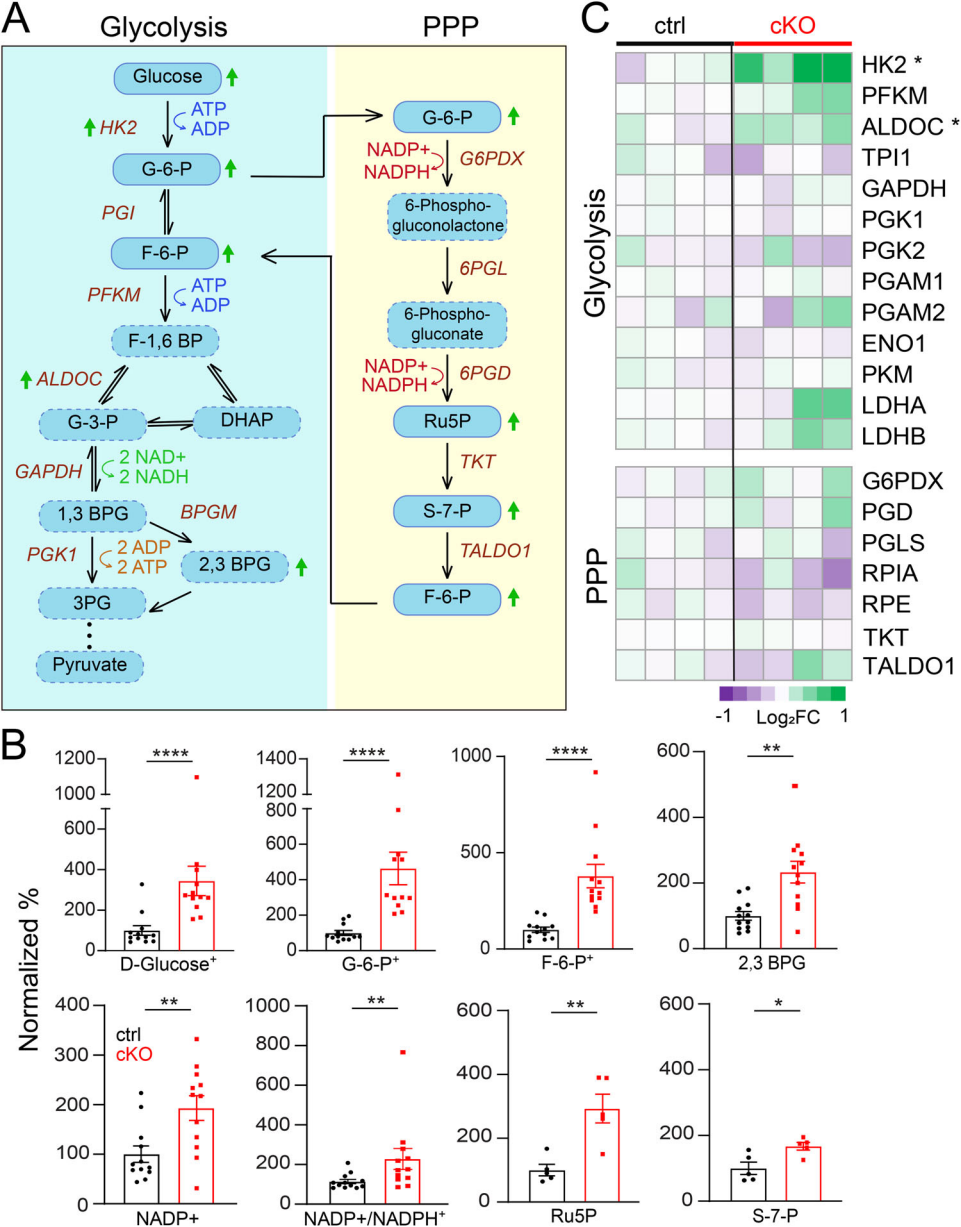
Neuronal NMNAT2 loss results in aberrant glucose metabolism. A) A diagram summarizes key proteins and metabolites involved in glycolysis and the pentose phosphate pathway (PPP). Arrows indicate significant changes in proteins or metabolites. Metabolites labeled with a dashed line were not detected in the MS analysis. B) Summaries for the normalized abundance of intermediate metabolites in glycolysis and PPP pathways significantly differ between ctrl and cKO cortices. Data from male and female mice were combined, as no significant sex differences were detected. (*n* = 6 per sex per group, except for Ru5P (*n* = 5 males); Student's t‐test, metabolites labeled with “+” were analyzed using the Mann–Whitney test). C) Heatmaps in log_2_‐transformed values summarize the proteomic abundance of enzymes involved in glycolysis and PPP (*n* = 4 males; Student's t‐test). All data are presented as mean +/‐SEM. *, *p*<0.05; **, *p*<0.01; ***, *p*<0.001; ****, *p*<0.0001. *See* Supporting Information *Statistics Table for detailed statistics*. Abbreviations: 1,3 BPG, 1,3‐bisphosphoglycerate; 2,3 BPG, 2,3‐bisphosphoglyceric acid; 3PG, 3‐phosphoglycerate; ALDOC, fructose‐bisphosphate aldolase C; DHAP, dihydroxyacetone phosphate; ENO1, Alpha‐enolase; F‐1,6 BP, fructose‐1,6‐bisphosphate; F‐6‐P, fructose‐6‐phosphate; G‐3‐P, glyceraldehyde‐3‐phosphate; G‐6‐P, glucose‐6‐phosphate; G6PDX, glucose‐6‐phosphate 1‐dehydrogenase X; GAPDH, glyceraldehyde‐3‐phosphate dehydrogenase; HK2, hexokinase‐2; LDHA, L‐lactate dehydrogenase; LDHB, L‐lactate dehydrogenase B chain; PFKM, ATP‐dependent 6‐phosphofructokinase; PGAM1, phosphoglycerate mutase 1; PGAM2, phosphoglycerate mutase 2; PGD, 6‐phosphogluconate dehydrogenase; PGK1, phosphoglycerate kinase 1, PGK2, phosphoglycerate kinase 2; PGLS, 6‐phosphogluconolactonase, PKM, pyruvate kinase; RPE, ribulose‐phosphate 3‐epimerase; RPIA, ribose‐5‐phosphate isomerase; Ru5P, ribulose‐5‐phosphate; S‐7‐P, sedoheptulose‐7‐phosphate; TALDO1, transaldolase; TKT, transketolase; TPI1, triosephosphate isomerase.

**Figure 4:**
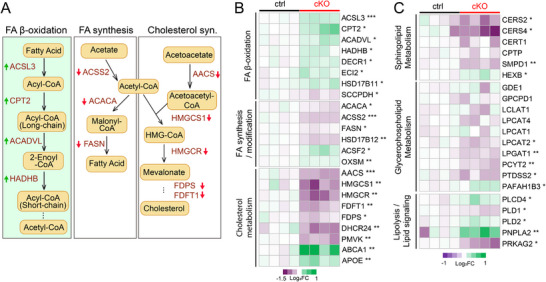
Neuronal NMNAT2 loss results in aberrant lipid metabolism. A) Diagrams summarizing proteins associated with lipolysis and lipogenesis pathways that are differentially expressed in cKO cortices. B) Heatmaps in log_2_‐transformed values summarize the proteomic abundance of enzymes in the FA synthesis/modification, FA β‐oxidation, and cholesterol synthesis pathways. Arrows indicate significant changes in proteins or metabolites. (*n* = 4 males; Student's *t*‐test) C) Heatmap summarizing log_2_ transformed fold changes of proteomic profiles associated with sphingolipid metabolism, glycerophospholipid metabolism, and lipolysis/lipid signaling. (*n* = 4 males; Student's *t*‐test). All data are presented as mean +/‐SEM. *, *p*<0.05; **, *p*<0.01; ***, *p*<0.001. See Supporting Information *Statistics Table for detailed statistics*. Abbreviations: AACS, acetoacetyl‐CoA synthetase; ABCA1, ATP binding cassette subfamily A member 1; ACACA, acetyl‐CoA carboxylase 1; ACADVL, very long‐chain specific acyl‐CoA dehydrogenase; ACSS2, acyl‐CoA synthetase short chain family member 2; ACSF2, medium‐chain acyl‐CoA ligase; ACSL3, fatty acid CoA ligase; APOE, Apolipoprotein E; CERT1, ceramide transfer protein; CERS2, ceramide synthase 2; CERS4, ceramide synthase 4; CPT2, carnitine palmitoyltransferase 2; CPTP, ceramide‐1‐phosphate transfer protein; DECR1, 2,4‐dienoyl‐CoA reductase; DHCR24, delta(24)‐sterol reductase; ECI2, enoyl‐CoA delta isomerase 2; FASN, fatty acid synthase; FDFT1, squalene synthase; FDPS, farnesyl pyrophosphate synthase; GDE1, glycerophosphodiester phosphodiesterase 1; GPCPD1, glycerophosphocholine phosphodiesterase; HADHB, trifunctional enzyme subunit beta; HEXB, beta‐hexosaminidase subunit beta; HMGCR, 3‐hydroxy‐3‐methylglutaryl‐coenzyme A reductase; HMGCS1, hydroxymethylglutaryl‐CoA synthase; HSD17B11, estradiol 17‐beta‐dehydrogenase 11; HSD17B12, very‐long‐chain 3‐oxoacyl‐CoA reductase; LCLAT1, lysocardiolipin acyltransferase 1; LPCAT1, lysophosphatidylcholine acyltransferase 1; LPCAT2, lysophosphatidylcholine acyltransferase 2; LPCAT4, lysophospholipid acyltransferase; LPGAT1, acyl‐CoA:lysophosphatidylglycerol acyltransferase 1; OXSM, 3‐oxoacyl‐(acyl‐carrier‐protein) synthase; PAFAH1B3, Platelet‐activating factor acetylhydrolase IB subunit alpha1; PCYT2, ethanolamine‐phosphate cytidylyltransferase; PLCD4, 1‐phosphatidylinositol 4,5‐bisphosphate phosphodiesterase delta‐4; PLD1, phospholipase D1; PLD2, phospholipase D2; PMVK, phosphomevalonate kinase; PNPLA2, patatin‐like phospholipase domain‐containing protein 2; PRKAG2, 5'‐AMP‐activated protein kinase subunit gamma‐2; PTDSS2, phosphatidylserine synthase 2; SCCPDH, saccharopine dehydrogenase‐like oxidoreductase; SMPD1, sphingomyelin phosphodiesterase.

To better understand how glucose and lipid metabolic pathways are affected by NMNAT2 loss in neurons, we combined the metabolomic and proteomic data sets to examine the key enzymes and intermediates in each pathway (**Figures** **3** and **4**). In NMNAT2 cKO brains, we found substantial increases in several glycolysis intermediates, including glucose, glucose‐6‐phosphate, fructose‐6‐phosphate, and 2,3‐bisphosphoglyceric acid (Figure 3A,B). Metabolites in the pentose phosphate pathway (PPP), such as ribulose‐5‐phosphate and sedoheptulose‐7‐phosphate, were also increased in the cKO cortices. Proteomic analysis identified increases in glycolytic enzymes, such as hexokinase 2 (HK2) and aldolase (ALDOC), in NMNAT2 KO cortices (Figure 3C). Such increases in glycolytic enzymes may reflect compensatory responses to the accumulation of upstream glycolytic intermediates. Overall, these results indicate that neuronal NMNAT2 loss results in significant alterations in glycolysis and the PPP pathways.

Regarding lipid metabolism, we found significant upregulation of key enzymes involved in FAO in NMNAT2 cKO tissue (Figure 4), such as carnitine palmitoyltransferase 2 (CPT2) and acyl‐CoA dehydrogenase (ACADVL). The ketogenesis enzymes, such as hydroxymethylglutaryl‐CoA synthase (HMGCS2) and D‐beta‐hydroxybutyrate dehydrogenase (BDH1), are upregulated in cKO cortices (Figure S1A,B). Together with the increased levels of 3‐hydroxybutyric acid (BHB), a ketone body, our data suggest that ketogenesis is enhanced in NMNAT2 cKO cortices (Figure S1C). Concurrently, several proteins involved in lipogenesis and cholesterol synthesis pathways, including acetyl‐CoA carboxylase alpha (ACACA) and acetoacetyl‐CoA synthetase (AACS), were significantly downregulated (Figure 4A,B). Additionally, proteins involved in lipid transport, such as ATP‐binding cassette subfamily A member 1 (ABCA1) and Apolipoprotein E (APOE), were upregulated in cKO cortices. In summary, our data indicate a metabolic shift in NMNAT2 cKO cortices towards FAO and ketogenesis, while glycolysis and lipolysis were significantly reduced.

### 2.4 NMNAT2 Loss in Neurons Triggers Massive Lipid Catabolism and Complete SARM1 Deletion Restores Its Lipid Levels

**Figure 5:**
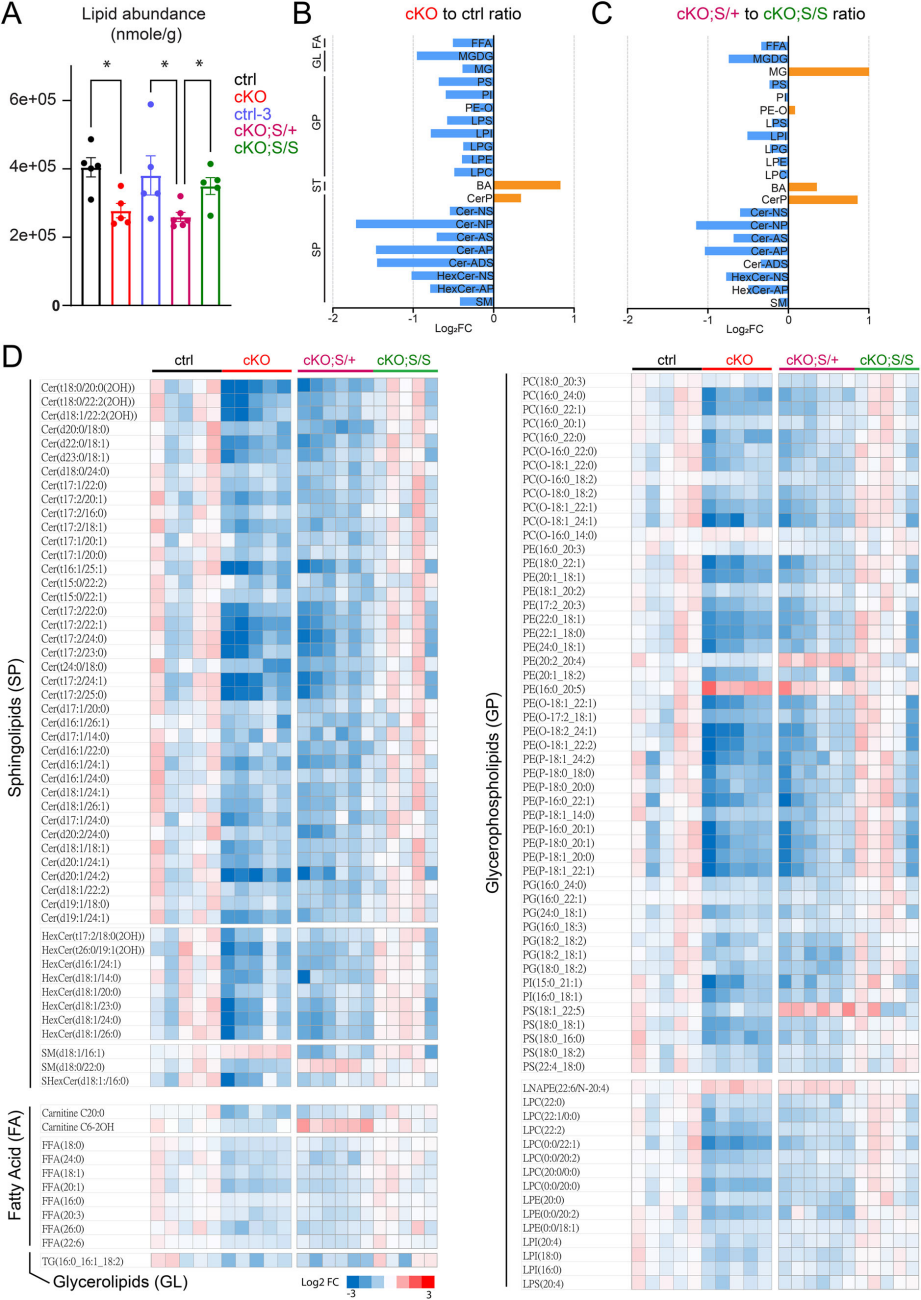
NMNAT2 loss results in a significant reduction in lipid abundance, and complete SARM1 deletion restores the lipid levels. A) Summary of the lipid abundance for control, cKO, control‐3, cKO;S^null/+^ (labeled in panels as cKO;S/+), and cKO;S^null/null^ (labeled in panels as cKO;S/S) cortices. (*n* = 5 males, except for cKO;S/+ (*n* = 6 males); ANOVA). B,C) Summary of log_2_‐transformed fold changes for the most highly differentially expressed lipid subclasses. D) Heatmaps highlight the top‐ranked differences in lipid content between groups. (*n* = 5 males for ctrl, cKO, and cKO;S/+; *n* = 6 males for cKO;S/S). Data are presented as mean +/‐SEM.*, *p*<0.05; **, *p*<0.01). *See* *Supporing Information* *Statistics Table for detailed statistics*. Abbreviations: BA, Bile Acid; Cer‐ADS, Ceramide Alpha‐Hydroxy Fatty Acid‐Dihydrosphingosine; Cer‐AP, Ceramide Alpha‐Hydroxy Fatty Acid‐Phytosphingosine; Cer‐AS, Ceramide Alpha‐Hydroxy Fatty Acid‐Sphingosine; Cer‐NP, Ceramide Non‐Hydroxy Fatty Acid‐Phytosphingosine; Cer‐NS, Ceramide Non‐Hydroxy Fatty Acid‐Sphingosine; CerP, Ceramide‐1‐phosphate; FA, Fatty Acids; FFA, Free Fatty Acid; GL, Glycerolipids; GP, Glycerophospholipids; HexCer‐AP, Hexosylceramide Alpha‐Hydroxy Fatty Acid‐Phytosphingosine; HexCer‐NS, Hexosylceramide Non‐Hydroxy Fatty Acid‐Sphingosine; LPC, Lysophosphatidylcholine; LPE, Lysophosphatidylethanolamine; LPG, Lysophosphatidylglycerol; LPI, Lysophosphatidylinositol; LPS, Lysophosphatidylserine; MG, Monoacylglycerol; MGDG, Monogalactosyldiacylglycerol; PE‐O, Ether‐linked Phosphatidylethanolamine; PI, Phosphatidylinositol; PS, Phosphatidylserine; SM, Sphingomyelin; SP, Sphingolipids; ST, Sterol lipids.

The prominent changes in proteins involved in fatty acid metabolism observed in NMNAT2 cKO cortices (Figure 4) prompted us to conduct a quantitative lipidomic analysis. Principal component analysis (PCA) showed clear separations between P16‐21 cKO and control cortices (Figure S2A). Strikingly, we found a substantial reduction in total lipid abundance in cKO cortices compared to control (**Figure** **5**A). Among the 1254 lipid species identified, 408 were significantly altered in the cKO cortices, with 382 being downregulated (Figure S2B). Several subgroups of sphingolipids and glycerophospholipids were downregulated in the cKO cortices (Figure 5B,D; Table S1). Only a few lipid subgroups, such as bile acids and ceramide phosphates, are elevated in cKO cortices. Notably, these lipidomic alterations correlate with reduced expression of key enzymes involved in glycerophospholipid and sphingolipid metabolism in cKO cortices (Figure 4C). For instance, ceramide synthases (CERS2, CERS4) and sphingomyelin phosphodiesterase (SMPD1) are downregulated, along with Lysophosphatidylglycerol Acyltransferase 1 (LPGAT1), Lysophosphatidylcholine Acyltransferase 2 (LPCAT2), Phosphatidylserine Synthase 2 (PSS2), and Phospholipase D1 (PLD1).

We and others have shown that genetically deleting SARM1 protects against the axonopathy caused by NMNAT2 loss.^[^ ^13^, ^25^, ^28^, ^29^ ^]^ Here, we aim to investigate whether deleting SARM1 can mitigate the metabolic reprogramming that occurs after neuronal NMNAT2 loss and subsequently restore brain health. NMNAT2 cKO mice were crossed with SARM1 knockout mice (S^null/null^) to generate NMNAT2 cKO mice in heterozygous (cKO;S^null/+^) or homozygous (cKO;S^null/null^) SARM1‐null backgrounds. Cortices from cKO;S^null/+^, cKO;S^null/null^, and littermate control mice from this breeding strategy (named as control‐2 to be distinguished from cKO littermate control despite the same genotype as Nex^cre/+^;NMNAT2^f/+^;S^+/+^) were collected for quantitative lipidomic analysis. We have found that the brains of control‐2 and cKO;S^null^/S^null^ mice appear morphologically normal, whereas degenerative morphologies are observed in the brains of cKO and cKO;S^null/+^ mice.^[^ ^13^ ^]^ The lipidomic analysis shows that the total lipid abundances in cKO;S^null/+^ cortices are significantly reduced, similar to the levels detected with cKO cortices (Figure 5A). In contrast, control‐3 and cKO;S^null/null^ cortices show normal lipid levels comparable to cKO littermate controls.

In cKO;S^null/+^ cortices, 234 lipids were significantly altered, with 204 downregulated compared to cKO;S^null/null^ cortices (Figure S2C,D). Several subgroups of sphingolipids and glycerophospholipids were downregulated, while bile acids and ceramide phosphates were upregulated in the cKO;S^null/+^ cortices compared to cKO;S^null/null^ cortices (Figure 5C,D). The most similar lipid changes in abundance for cKO and cKO;S^null/+^ compared to control and cKO;S^null/null^, respectively, are bile acids and lipids within the sphingolipid and sterol lipid subclasses (Table S1). KEGG pathway analysis with altered lipid species reveals the common changes in many pathways, including glycerophospholipid metabolism, sphingolipid metabolism, retrograde endocannabinoid signaling, insulin resistance, necroptosis, and autophagy (Table S2). In summary, our analysis reveals that, upon the complete deletion of SARM1, many lipid alterations in cKO cortices become normalized.

### 2.5 NMNAT2 Loss Alters Nucleotide and Glutathione Metabolism, While SARM1 Deletion Ameliorates These Changes

**Figure 6:**
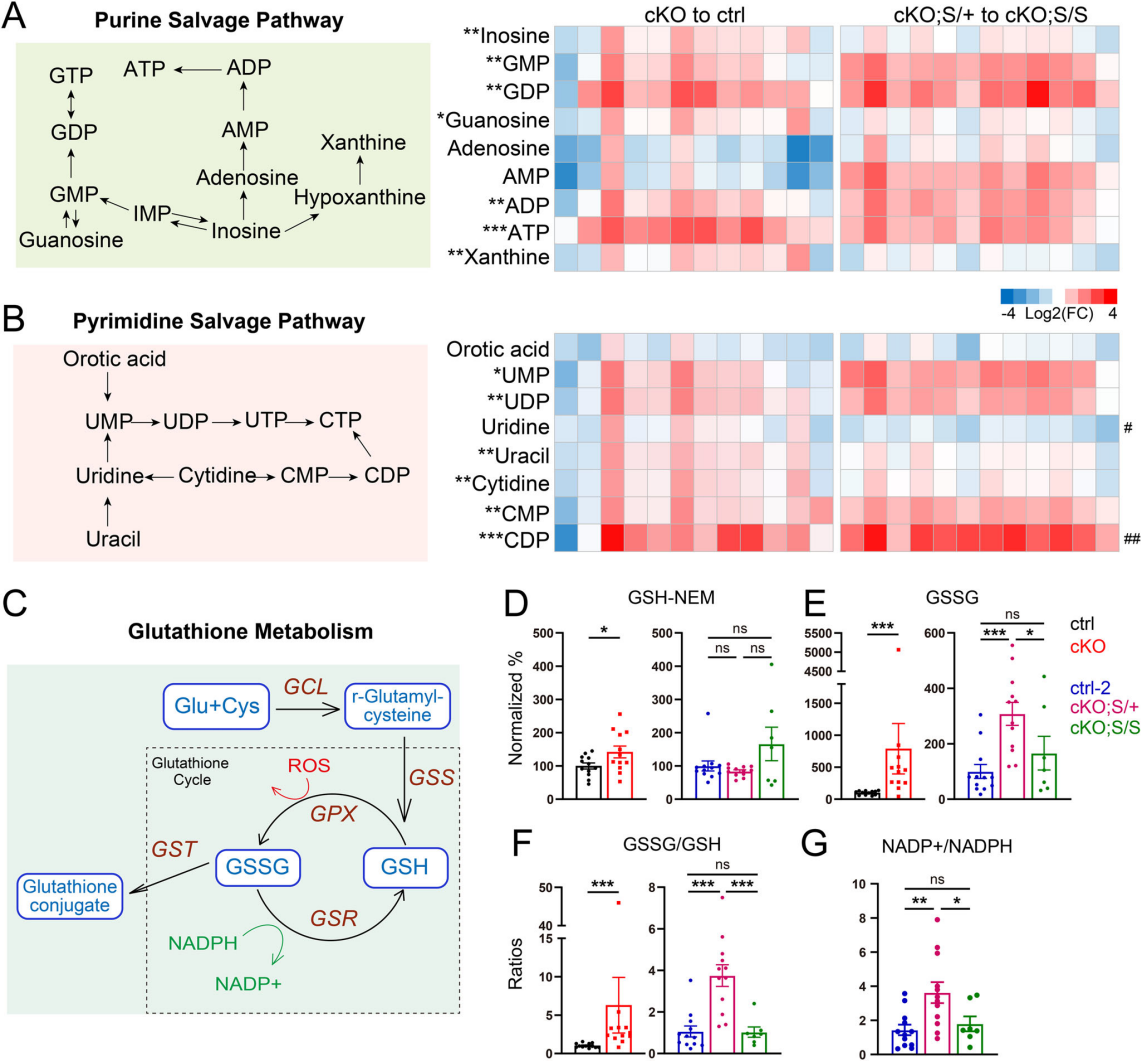
Comparative analysis of nucleotide salvage and redox metabolic pathways in NMNAT2 and SARM1 mutant cortices. A,B) NMNAT2 loss disrupts the nucleotide salvage pathways and complete SARM1 deletion restores the pathways. A) Schematic representation of the purine salvage pathway and log_2_‐transformed heatmaps summarizing fold changes in intermediate metabolite levels for cKO to ctrl and cKO;S/+ to cKO;S/S. B) A diagram of the pyrimidine salvage pathway and log_2_‐transformed heatmaps summarizing fold changes in intermediate metabolites. Asterisks “*” indicate comparisons between control and cKO, *n* = 6 per sex per group (Student's *t*‐test). The hash symbols “#” indicate comparisons between cKO;S/+ and cKO;S/S, *n* = 6 per sex per group for cKO;S/+, *n* = 3 males and 4 females for cKO;S/S (Kruskal–Wallis test). (C) Glutathione metabolism diagram. D) Summary of normalized GSH percentages. For the NMNAT2 cKO group, *n* = 6 per sex per group (Student's *t*‐test). For the NMNAT2–SARM1 double transgenic groups, *n* = 6 per sex per group for Ctrl‐2 and cKO;S/+, and *n* = 3 males and 4 females for cKO;S/S (Kruskal–Wallis test). E) Summary of normalized GSSG percentages. For the NMNAT2 cKO group, *n* = 6 per sex per group (Mann–Whitney test). For the NMNAT2–SARM1 double transgenic groups, *n* = 6 per sex per group for Ctrl‐2 and cKO;S/+, and *n* = 3 males and 4 females for cKO;S/S (ANOVA). F) Summary of GSSG/GSH ratios. For the NMNAT2 cKO group, *n* = 6 per sex per group (Mann–Whitney test). For the NMNAT2–SARM1 double transgenic groups, *n* = 6 per sex per group for Ctrl‐2 and cKO;S/+, and n = 3 males and 4 females for cKO;S/S (Kruskal–Wallis test). G) Summary of NADP+/NADPH ratio. *n* = 6 per sex per group for Ctrl‐2 and cKO;S/+, and *n* = 3 males and 4 females for cKO;S/S (ANOVA). All data are presented as mean +/‐SEM. *, p<0.05; **, p<0.01; ***, p<0.001. *See* *Supporting Information* *Statistics Table for detailed statistics*.

LC‐MS analysis targeting redox metabolites and nucleotides was conducted with the cortices prepared from control‐2, cKO;S^null/+^, and cKO;S^null/null^ mice. Surprisingly, no difference in NAD^+^/NADH ratios between cKO;S^null/+^ and control‐2 or cKO;S^null/null^ cortices (ratios: control‐2, 1.12±0.15; cKO;S^null/+^, 0.91 ± 0.07; cKO;S^null/null^, 0.84±0.10) were observed. However, we found significant changes in several metabolites within the purine and pyrimidine salvage pathways in cKO;S^null/+^ compared to control‐2 or cKO;S^null/null^ cortices (**Figure** **6**A,B). Similar changes in purine/pyrimidine metabolite abundance were also identified in cKO cortices compared to controls. NAD^+^ regulates nucleotide metabolism,^[^ ^40^ ^]^ and research shows that NAD^+^ impairment alters the purine and pyrimidine metabolic pathways.^[^ ^42^ ^]^ Thus, despite the normalization of NAD^+^/NADH ratios upon the deletion of one copy of SARM1, there are likely to be significant deficits in local NAD^+^ homeostasis to perturb the purine and pyrimidine pathways in cKO;S^null/+^, while complete SARM1 deletion ameliorates the deficits.

Our targeted LC‐MS analysis also revealed elevated ratios of oxidized glutathione (GSSG) to reduced glutathione (GSH) in cKO;S^null/+^ compared to control‐2 or cKO;S^null/null^ cortices (Figure 6C–F). Significant increases in GSSG/GSH (Figure 6F) ratios were also observed in cKO cortices compared to controls. Glutathione is the most abundant non‐protein thiol in mammalian cells, and it acts as a vital reactive oxygen species (ROS) scavenger crucial for cellular defense against oxidative stress.^[^ ^43^, ^44^, ^45^, ^46^ ^]^ The conversion of GSSG back to GSH, essential for maintaining redox balance, is facilitated by NADPH through the catalysis of glutathione reductase (GR).^[^ ^45^, ^47^ ^]^ Interestingly, NADP^+^/NADPH ratios are elevated in cKO and cKO;S^null/+^ cortices compared to control, control‐2, or cKO;S^null/null^ cortices, respectively (Figures 3B and 6G). The increased NADP+/NADPH ratios may impair catalysis by GR, consequently increasing GSSG/GSH ratios.

### 2.6 Loss of Neuronal NMNAT2 Triggers Inflammatory Responses and Motor Behavior Deficits, Which are Mitigated by SARM1 KO

**Figure 7:**
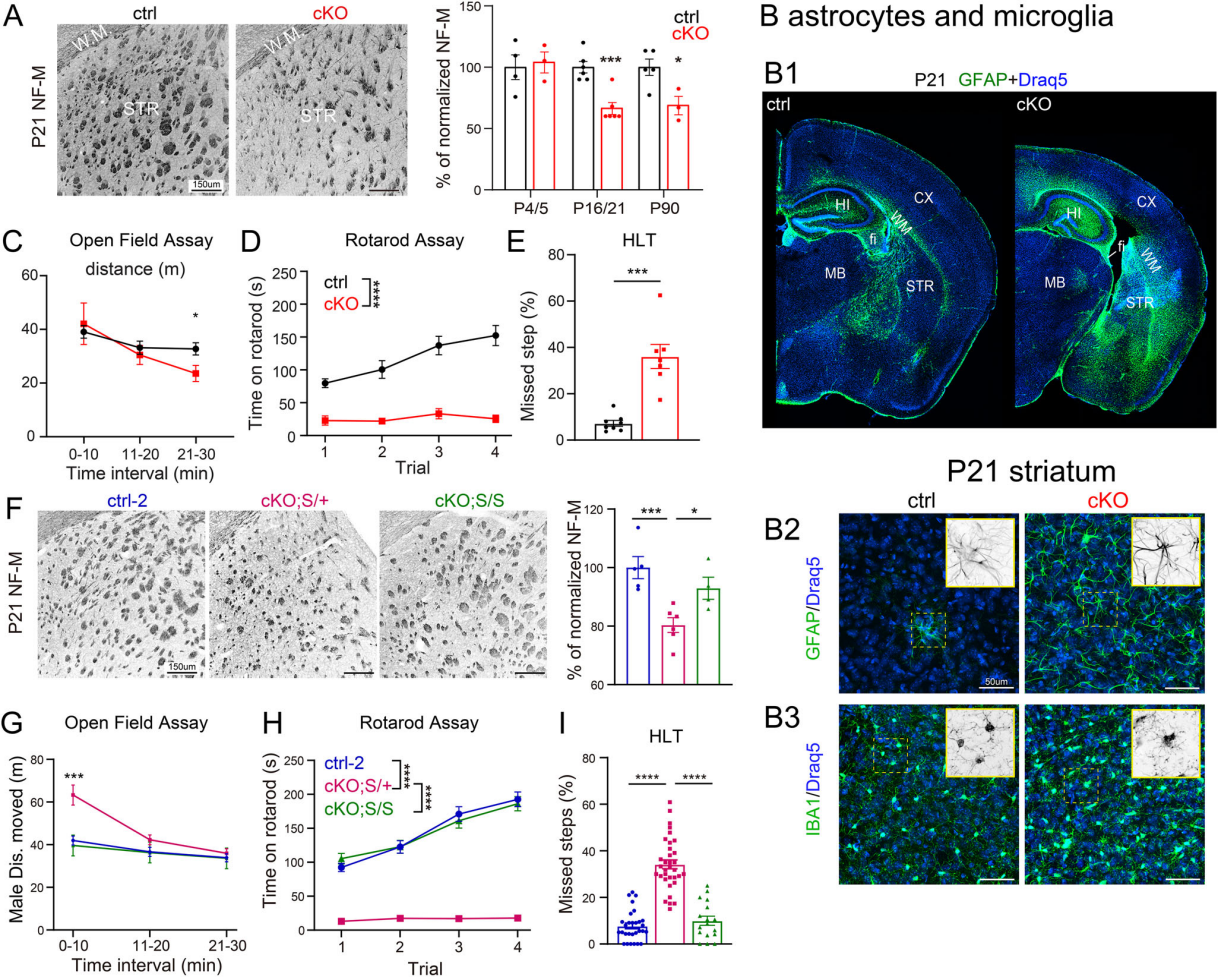
Deleting NMNAT2 in glutamatergic neurons leads to axonal loss, inflammation, and motor deficits, while complete SARM1 deletion improves the neurodegeneration phenotypes. A) Representative NF‐M immunostaining images reveal axonal tracts in the striatum (left panels). Bar graphs (right panel) summarize normalized NF‐M pixel densities detected in the striatum at P4/5 (ctrl, *n *= 4; cKO *n* = 3), P16/21 (ctrl, *n* = 6; cKO *n *= 6), and P90 (ctrl, *n* = 5; cKO, *n* = 3); Student's t‐test. B1–B4) Stitched confocal GFAP staining images (Draq 5 labels nuclei) of coronal sections from ctrl and cKO brains B1). B2) Representative GFAP staining images from P21 ctrl and cKO striatum. B3) Representative IBA1 staining images. C) Summary for total distance traveled by ctrl and cKO mice in the open field arena during the 30‐min session (ctrl, n=11; cKO *n* = 8); Student's *t*‐test. D) Summary of time on rotarod of ctrl and cKO mice during the accelerating rotarod test (ctrl, *n* = 9; cKO, *n* = 8); Student's *t*‐test. E) Summary for the percentage of missed steps during horizontal ladder walking test (HLT) (ctrl, *n* = 8; cKO, *n* = 7); Mann–Whitney test. F) Representative NF‐M immunostaining images in the striatal region of P21 control‐2 (ctrl‐2; see Methods for details), cKO;S/+, and cKO;S/S brains, along with the summary of normalized NF‐M levels. ctrl‐2, *n* = 5; cKO;S/+, *n* = 6; cKO;S/S, *n* = 4; AVOVA. G–I) Summaries for OFA (G; ctrl‐2, *n* = 15; cKO;S/+, *n* = 15; cKO;S/S, *n* = 8; Student's *t*‐test.), rotarod (H; ctrl‐2, n=30; cKO;S/+, *n* =33; cKO;S/S, *n* = 18; Student's *t*‐test.) and HLT tests (I; ctrl‐2, *n*=29; cKO;S/+, *n*=34; cKO;S/S, *n*=17; AVOVA). All data are presented as mean +/‐SEM. *, *p*<0.05; **, *p*<0.01; ***, *p*<0.001; ****, *p*<0.0001. *See* *Supporting Information* *Statistics Table for detailed statistics*. Abbreviations: CX, cortex; HI, hippocampus; WM, white matter; DG, dentate gyrus; STR, striatum; fi, fimbria; MB, midbrain.

Our proteomic data showed higher abundances of GFAP (a marker for reactive astrocytes),^[^ ^48^, ^49^ ^]^ CD68 (a microglia marker^[^ ^50^ ^]^), and other glia‐specific proteins (Table S3) in NMNAT2 cKO cortices. Additionally, several complement system components and IL‐18, a pro‐inflammatory cytokine, are also increased in cKO cortices. These proteomic data indicate inflammatory responses in NMNAT2 cKO brains. Inflammation and axonal loss are hallmarks of many neurodegenerative diseases.^[^ ^51^, ^52^ ^]^ To examine when NMNAT2 loss results in neurodegenerative phenotypes, immunostainings for GFAP, IBA1 (a microglia marker^[^ ^53^ ^]^), and neurofilament‐M (NF‐M; a marker for axons) were conducted in coronal brain sections prepared from P5, P16/21, and P90 control and cKO mice. Similar to our previous findings, NF‐M+ axon densities at P5 are similar in cKO and control groups in the white matter and striatum, where long‐range cortical axons traverse the basal ganglia (**Figure** **7**A and ref. [13]). However, after P16/21, NF‐M+ axons are significantly reduced. Elevated GFAP signals peaked at P16/21 in the hippocampus, fimbria, white matter, and striatum of cKO mice (Figure 7B1,B2 and Figure S3A‐B). GFAP+ astrocytes in cKO brains exhibited bushy morphologies with thickened processes (Figure 7B2 and Figure S3C), a characteristic feature of reactive astrocytes during inflammation.^[^ ^54^ ^]^ Additionally, we observed a significant increase in the densities of IBA1+ microglia in the cKO hippocampus and in the cKO striatum from P5 to P90 (Figure 7B3 and Figure S4A,B). Microglia in cKO brains often had large somas with thicker but shorter processes (Figure 7B3 and Figure S4C), which is a characteristic of reactive microglia.^[^ ^55^ ^]^

Next, we examined if adult NMNAT2 cKO mice exhibit motor behavior deficits in the open‐field assay (OFA), the accelerating rotarod test, and the horizontal ladder walk test (Figure 7C–E). The cKO mice exhibited relatively normal locomotion for the first 20 min in the OFA (Figure 7C). However, they had significant difficulties in staying on the accelerating rotarod, as indicated by markedly reduced time on the rotarod (Figure 7D). In contrast to control mice, which had very few missed steps while walking on the horizontal ladder, cKO mice missed significantly more steps (Figure 7E). These motor behavioral impairments noted in cKO mice suggest deficits in their motor coordination.

cKO;S^null/+^ mice also displayed reduced NF‐M+ axon densities (Figure 7F) and increased GFAP and IBA1 immunoreactivity in the striatum compared to control‐2 and cKO;S^null/null^ mice (Figure S5). Excitingly, the complete absence of SARM1 alleviates motor coordination deficits observed in NMNAT2 cKO mice (Figure 7H‐I), while cKO;S^null/+^ mice show hyperlocomotion during the first 10 min in OFA (Figure 7G), severe impairments in the accelerating rotarod (Figure 7H) and horizontal ladder walk (Figure 7I) tests. Collectively, the immunostaining and behavioral data suggest that the total loss of SARM1 ameliorates the neuroinflammation, axonopathy, and motor coordination deficits caused by NMNAT2 deficiency.

### 2.7 Lipid Metabolism‐Related Proteome Module Reversed by SARM1 KO is Linked to Neurodegenerative Phenotype

**Figure 8:**
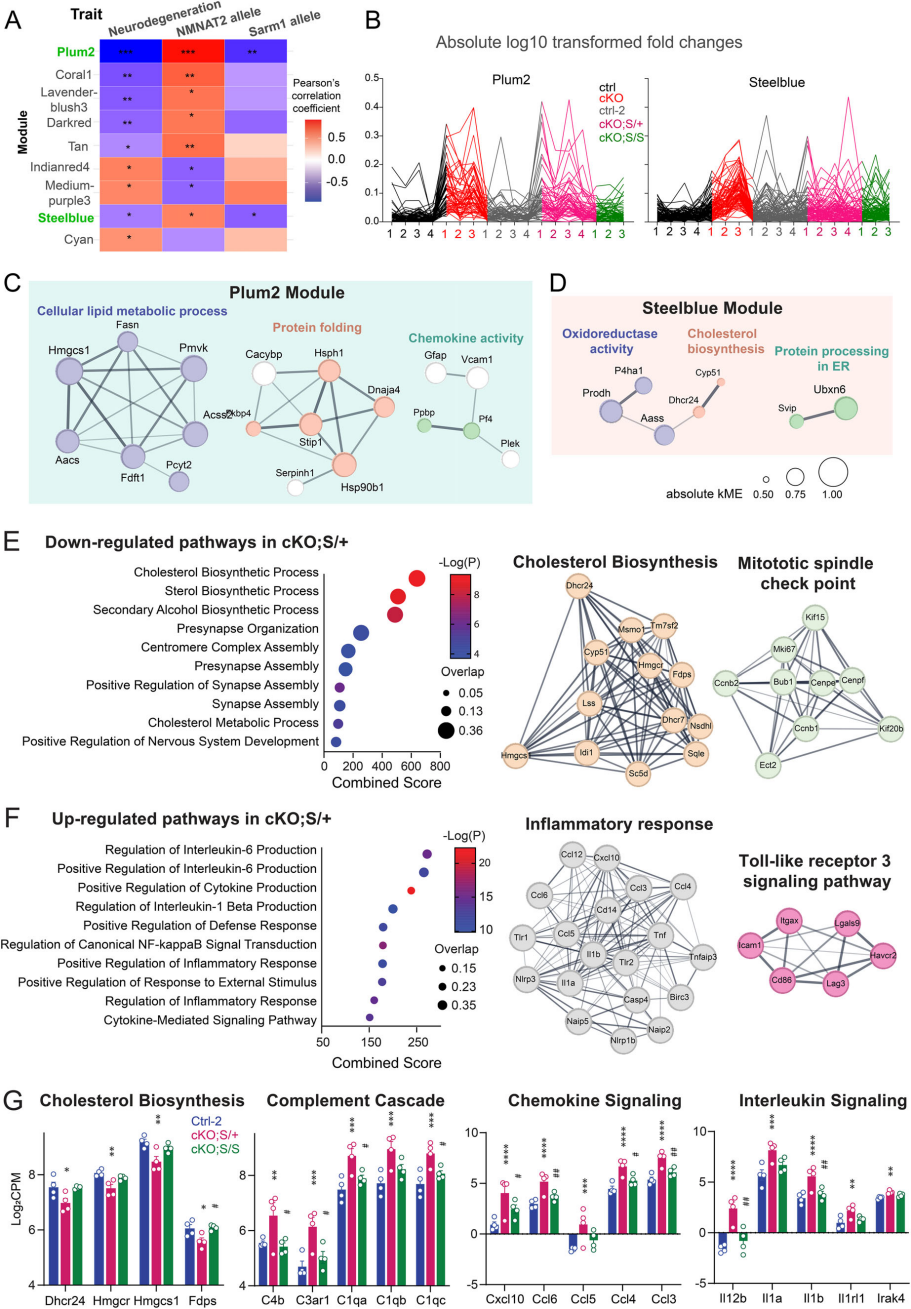
Bioinformatic analysis with WGCNA identified several protein modules that significantly correlated with neurodegenerations and NMNAT2 abundance. SARM1's presence exerts similar impacts as neurodegeneration on two protein modules. A) Module‐trait correlation heat map. Color denotes the Pearson correlation coefficient. *p*<0.05 *, *p*<0.01 **, *p*<0.001 *** by student's *t*‐test. B) Individual protein expression patterns across samples within the Plum2 and Steelblue module. Absolute log10‐transformed fold changes were derived from the original protein abundance value before batch‐effect reduction. C,D) PPI network of Plum2 C) and Steelblue D) module proteins, with its edge thickness representing the interaction score and its node size showing the eigenprotein‐based connectivity (kME), which is the Pearson correlation between the expression profile of a gene and the first principal component of the expression matrix of all proteins within the module (eigenprotein). E,F) Summary of GO pathway enrichment (left panels) and STRING interaction network analyses (right panels) from sorted astrocytes, showing genes upregulated E) and downregulated F) in cKO;S/+ compared to control astrocytes. In the bubble plots, bubble size represents the overlap ratio of genes associated with each pathway, and the x‐axis indicates the combined pathway score. The right panels display key hub genes identified by STRING, with edge thickness corresponding to the interaction confidence score. Sample size: *n* = 4 per genotype. G) Summary of log_2_CPM values for genes involved in multiple pathways from RNA‐seq analysis of sorted astrocytes. Asterisks “*” indicate comparisons between control and cKO;S/+, and hash symbols “#” indicate comparisons between cKO;S/+ and cKO;S/S. *n* = 4 per genotype. Bar plots show mean ± SEM log_2_CPM values for each genotype. Statistical significance was determined from differential expression analysis using the edgeR generalized linear model with Benjamini–Hochberg false discovery rate (FDR) correction. *See* *Supporting Information* *Statistics Table for detailed statistics*.

To gain molecular insight into the rescue offered by complete SARM1 deletion on neurodegeneration caused by loss of NMNAT2 in the cKO cortices, we performed proteomic profiling with control‐2, cKO;S^null/+^, and cKO;S^null/null^ cortices. After batch effect reduction and integration with the proteomic datasets from control and cKO cortices shown above (Figure 2), 6850 proteins commonly detected in all datasets, including control, cKO, control‐2, cKO;S^null/+^, and cKO;S^null/null^, were used to conduct the weighted gene correlation network analysis (WGCNA). Proteins exhibiting a similar expression pattern across samples were clustered into individual modules. Among the total 36 modules identified (Figure S6), we identified 8 modules that show significant correlations with neurodegenerative phenotypes and NMNAT2 allele dosages (**Figure** **8**A). Interestingly, only two of these 8 modules exhibited expression patterns that were significantly reversed by SARM1 allele dosage (Figure 8A,B). A single‐allele deletion of SARM1 readily rescued protein expression in the Steelblue module, whereas complete loss of SARM1 reversed protein changes in the Plum2 module. Interpreted by the STRING PPI network, the Plum2 module is chiefly enriched in cellular lipid metabolic process and protein folding, followed by chemokine activity (Figure 8C). AACS, HMGCS1, FDFT1, and PMVK are involved in cholesterol metabolism (Figure 4 and Table S3), ACSS2 and FASN mediate fatty acid synthesis, and PCYT2 catalyzes the second step of phosphatidylethanolamine (PE) synthesis. STRING PPI network analysis with proteins in the Steelblue module identifies proteins involved in oxidoreductase activity, cholesterol biosynthesis, and protein processing in the ER (Figure 8D). The WGCNA outcome, along with our lipidomic data, shows that SARM1 deletion alleviates lipid metabolic deficits positively associated with the neurodegenerative phenotype and negatively associated with the NMNAT2 allele.

Many proteins in lipid metabolism identified in Plum 2 and Steelblue modules are known to be astrocyte‐enriched, e.g., AACS, HMGCS1, FDFT1, ACSS2, and FASN. This finding encouraged us to examine transcriptomic profiles of astrocytes isolated from the brains of P16 control‐2, cKO;S^null/+^, and cKO;S^null/null^ mice. Using the Magnetic Activated Cell Sorting (MACs) method, ∼10^5^–10^6^ astrocytes were isolated per mouse brain (see Experimental Section). mRNAs were purified and subjected to RNAseq analysis. The high expression of astrocyte marker genes, along with the relatively low expression of neuron or microglia marker genes, confirms the astrocyte enrichment with the MACs method (Figure S7F). Robust transcriptomic changes were detected in cKO;S^null/+^ astrocytes (1219 upregulated and 380 downregulated versus control) (Figure S7). GO analyses of these differentially expressed genes revealed a significant downregulation in cholesterol biosynthesis, accompanied by upregulation in immune and inflammatory pathways in cKO;S^null/+^ astrocytes (Figure 8E–G). Similar pathway changes were also identified in the proteomic analyses conducted with whole cortices (Figure 8C‐D). Suggesting that astrocytes contribute to these bulk proteomic changes. The minimal transcriptomic differences between cKO;S^null/null^ and control astrocytes (33 up /15 down versus control) indicate that SARM1 deletion prevents the impact of NMNAT2 KO in neurons on astrocytes. These data highlight the SARM1‐dependent impact on astrocytes of neuronal NMNAT2 loss.

To further assess whether NMNAT2 KO neurons affect wild‐type (WT) astrocytes and disrupt neuron–astrocyte metabolic cooperation, we examined mitochondrial respiration in neuron‐only versus neuron–astrocyte co‐cultures. Primary cortical neurons were isolated from NMNAT2 WT and KO E15.5 embryos as previously described.^[^ ^13^ ^]^ WT astrocytes were added to half of the neuronal cultures at day‐in‐vitro 2 (DIV2). Four culture conditions were prepared: (1) WT neurons, (2) NMNAT2 KO neurons, (3) WT neurons co‐cultured with WT astrocytes, and (4) KO neurons co‐cultured with WT astrocytes. Mitochondrial respiration was assessed at DIV8, DIV10, and DIV12 using the Agilent Seahorse Mito Stress Test. Oxygen consumption rates (OCRs) were measured to determine basal, ATP‐linked, and maximal respiration rates following sequential injections of oligomycin, FCCP, and rotenone + antimycin A.

In neuron‐only cultures, NMNAT2 KO neurons, in comparison to WT neurons, showed a consistent reduction in basal, ATP‐linked, and maximal respiration at DIV8–10 but exhibited normal respiration by DIV12 (Figure S8A–C). In neuron–astrocyte co‐cultures, KO neuron‐containing cultures exhibited progressive deterioration of mitochondrial respiration, whereas WT co‐cultures remained stable across DIV8–12. At DIV8, astrocyte addition enhanced maximal respiration in both WT and KO neuron‐containing co‐cultures (Figure S8C). By DIV10, however, co‐culturing KO neurons with WT astrocytes led to a pronounced decline in respiration compared with KO neurons alone. When we compared the percentages of OCR changes normalized to the basal values (Figure S8E–F), the most notable difference between KO and WT neurons in the presence of astrocytes was the progressive decline in maximal respiration from DIV8 to DIV12. No difference was observed between WT and KO neurons in baseline normalized OCRs. These data suggest that the impairment of mitochondrial spare capacity occurs in cultures containing KO neurons and WT astrocytes, and the presence of astrocytes exacerbates the energetic failure.

## 3 Discussion

Maintaining NAD^+^ homeostasis is critical to support brain energy metabolism and health. NMNAT2 is the most abundant NAD^+^‐synthesizing enzyme in cortical neurons^[^ ^12^ ^]^ and is required for neuronal health.^[^ ^13^, ^14^, ^56^ ^]^ Its loss or reduction activates SARM1, a NAD^+^ hydrolase, leading to further NAD^+^ depletion and axon degeneration.^[^ ^21^, ^22^, ^28^ ^]^ Here, we utilized mice lacking NMNAT2 in post‐mitotic glutamatergic neurons to examine how NAD^+^ reduction in neurons affects energy homeostasis in the cerebral cortex. Multi‐omics analyses revealed extensive proteomic, metabolomic, and lipidomic remodeling upon NMNAT2 loss, including a marked shift from glucose to fatty acid catabolism, diminished lipid synthesis, and accumulation of sugars and related metabolites. NMNAT2 deficiency also altered astrocytic transcriptomic profiles, particularly in pathways related to cholesterol biosynthesis and inflammation. Notably, complete SARM1 deletion restored lipid abundance, normalized glial reactivity, and prevented neurodegenerative phenotypes in NMNAT2 KO mice. SARM1 deletion restores the transcriptomic profiles of astrocytes. These findings indicate that NAD⁺ depletion in neurons induces SARM1‐dependent maladaptive metabolic reprogramming that predisposes the brain to inflammation and neurodegeneration.

### 3.1 Impact of NMNAT2 Loss on Metabolic Pathways and the Mitigating Effects of SARM1 Deletion

Our study suggests that NMNAT2 loss in glutamatergic neurons triggers significant alterations in several metabolic pathways in the cortex, including nicotinate/nicotinamide, carbohydrate, lipid, nucleotide, and glutathione metabolisms. The observation of a substantial accumulation of sugar and sugar derivatives in NMNAT2 cKO brains was surprising, given the presence of other NMNATs and the specific deletion of NMNAT2 in glutamatergic neurons. The elevated glucose, glucose‐6‐phosphate, and fructose‐6‐phosphate levels, together with PPP intermediate metabolites, such as ribulose‐5‐phosphate and sedoheptulose‐7‐phosphate (Figure 3A,C), suggest elevated flux through the PPP in NMNAT2 cKO cortices.

The top pathways dysregulated upon NMNAT2 loss identified by proteomic analysis are primarily lipid metabolic pathways, including those involved in fatty acid synthesis/degradation/β‐oxidation and synthesis/degradation of ketone bodies (Figure 2C). Our lipidomic analysis revealed a substantial reduction of total lipid levels in NMNAT2 cKO cortices (Figure 5; Table S1). Many subgroups of sphingolipids and glycerophospholipids are down‐regulated in cKO cortices. Studies show that their metabolic processes are altered in various neurodegenerative disease conditions.^[^ ^57^, ^58^ ^]^ Proteomic data show that NMNAT2 cKO cortices had reduced levels of key lipid synthesis enzymes, such as ACSS2, ACACA, and FASN, while higher levels in key enzymes involved in FAO, such as ACADVL, ACSL3, and CPT2 (Figure 4), were increased. Together with the dramatic reduction in total lipid abundance, our data suggest that NMNAT2 loss decreases cortical lipogenesis while enhancing cortical lipid catabolism in the cortex. Lipid metabolism is fundamental for maintaining neurotransmission, cellular structure, and signaling.^[^ ^59^, ^60^, ^61^, ^62^ ^]^ Thus, the perturbed lipid metabolism upon NMNAT2 loss is likely to contribute to many of the phenotypes detected with proteomic analysis, histology, and behavioral assays.

The brain's high energy demand is primarily met by metabolizing glucose.^[^ ^63^, ^64^, ^65^ ^]^ It has a limited lipid storage capacity and depends on tightly regulated lipid synthesis, turnover, and utilization to maintain homeostasis.^[^ ^2^, ^33^, ^34^, ^66^ ^]^ However, brain metabolism is plastic, and the brain can use alternative fuel sources in response to metabolic stresses such as starvation and glycolytic impairment.^[^ ^31^, ^67^, ^68^, ^69^, ^70^ ^]^ In this regard, ketones such as acetoacetate and β‐hydroxybutyrate (BHB) represent an important alternative energy source for the brain. While ketogenesis predominantly occurs in hepatocytes,^[^ ^70^, ^71^ ^]^ in vitro studies suggest that astrocytes can synthesize ketone bodies due to their ability to oxidize fatty acids.^[^ ^32^, ^70^, ^72^ ^]^ Our findings support this hypothesis by revealing that NMNAT2 cKO cortices exhibit an increased abundance of the ketogenesis‐related enzymes, HMGCS2 and BDH1, as well as elevated levels of the ketone BHB (Figure S1), which was recently shown to be metabolized by neurons in a cell‐autonomous manner to fuel synaptic transmission.^[^ ^37^ ^]^ Thus, our proteomic data suggest an enhanced ketogenesis in NMNAT2 KO cortices, but we cannot rule out an increase of ketones from peripheral sources.

Our proteomic studies found that ABCA1 and APOE abundance are upregulated in NMNAT2 cKO cortices. APOE is the main protein component of lipoprotein particles produced by astrocytes and microglia, and it mediates various aspects of lipid homeostasis in the brain.^[^ ^73^, ^74^, ^75^, ^76^ ^]^ ABCA1 is the primary contributor of lipids to APOE‐lipoproteins in the extracellular space.^[^ ^77^ ^]^ Such lipidated APOE‐lipoproteins supply neurons with cholesterol and phospholipids from astrocytes, while also shuttling lipid peroxides from neurons to astrocytes for detoxification. Although our lipidomic analysis did not detect changes in cholesterol levels, our proteomic analysis revealed reductions in several cholesterol‐synthesizing enzymes, such as AACS and HMGCS, in NMNAT2 cKO cortices (Figure 4).

Neurodegenerative phenotypes in NMNAT2 cKO mice are mitigated by genetic SARM1 deletion. Does the absence of SARM1 activation prevent the reduction in NAD levels and thus avoid the widespread metabolic reprogramming? We found that deleting one copy of SARM1 is sufficient to restore the cortical NAD^+^/NADH ratios. However, the reduced cortical lipid levels in the cortices and neurodegenerative phenotypes are similar between cKO;S^null/+^ and NMNAT2 cKO mice (Figures 5 and 7). It is possible that our metabolomic analysis with whole cortices failed to detect localized NAD^+^ metabolism deficits in cKO;S^null/+^ brains, and localized NAD^+^ metabolism deficits may be sufficient to cause the maladapted metabolic plasticity. Eliminating two copies of SARM1 in NMNAT cKO mice significantly restores lipid, purine/pyrimidine, and glutathione levels (Figure 6). Regarding lipid metabolism, the most notable reversal upon total SARM1 deletion is the abundance of lipid species in the sphingolipid and sterol lipid subgroups, as well as bile acids. Congruently, WCGNA analysis of proteomic profiling revealed a protein module that associates with cholesterol, fatty acid, and PE synthesis pathways is negatively correlated to NMNAT2 expression and positively correlated to neurodegenerative phenotypes and SARM1^wt^ alleles (Figure 8).

### 3.2 Reducing Neuronal NAD+ Levels Activates Glia

The metabolism of glia and neurons is extensively coupled.^[^ ^36^, ^78^ ^]^ Astrocytes are positioned between neurons and the vasculature to control energy fluxes and nutrient supplies required for neuronal function.^[^ ^79^ ^]^ Astrocytes become “reactive” upon metabolic changes in glucose and lipid metabolism,^[^ ^80^ ^]^ while microglia are activated by increases in extracellular purines, pyrimidines, or cytokines.^[^ ^81^, ^82^ ^]^ Our proteomic analysis reveals that neuronal NMNAT2 loss elevates inflammatory responses, evident by significant increases in reactive glia markers (Table S3), pro‐inflammatory cytokines, and several components of the complement system in the cKO mice.^[^ ^83^, ^84^ ^]^ Histological evaluations of GFAP and IBA1 immunostaining also found inflammatory changes in cKO brains. These are particularly prominent in white matter and in the striatum, which is enriched with long‐range axons projecting from glutamatergic neurons (Figure 7B). This observation suggests that signals originating from the axons of NMNAT2 KO glutamatergic neurons activate neighboring astrocytes and microglia. Our astrocyte RNAseq study reveals up‐regulation of interleukin 1β/6, cytokine production, NF‐κB signaling, and many inflammatory response pathways in cKO;S^null/+^ astrocytes compared to control or cKO;S^null/null^ astrocytes (Figure 8F,G). These data support the non‐cell‐autonomous effects of NMNAT2 KO neurons on astrocytes, highlighting SARM1's role in triggering astrocyte inflammation.

Given that astrocytes are the primary brain cells capable of efficiently oxidizing medium‐ and long‐chain fatty acids for energy,^[^ ^2^, ^85^, ^86^, ^87^, ^88^ ^]^ the upregulation of FAO enzymes and BHB (Figure S1) suggests the possibility that astrocytes in NMNAT2 cKO cortices are oxidizing fatty acids to supply neurons with ketone bodies. This is consistent with previous studies showing astrocytes upregulate their FAO and ketogenesis to sustain neuronal energy demands.^[^ ^65^, ^89^ ^]^ ACSF2, HMGCR, and PLD2, the upregulated enzymes involved in lipid metabolism in cKO cortices, are predominantly expressed in astrocytes.^[^ ^90^, ^91^ ^]^ In contrast, the abundance of fatty acid/cholesterol synthesis enzymes known to be enriched in astrocytes (e.g., ACSS2, FASN, HMCS1, and FDFT1, listed in Figure 4) was significantly reduced in cKO cortices. Astrocyte RNA‐seq analysis also found significant down‐regulation of key cholesterol biosynthetic genes, such as Hmgcr, Hmgcs1, Fdps, and Dhcr24 in cKO;S^null/+^ astrocytes compared to control (Figure 8G). Such differences were abolished when SARM1 was deleted entirely. Together, our data suggest that lipid metabolism in astrocytes near NMNAT2 KO neurons is reprogrammed in a SARM1‐dependent manner.

Additionally, our in vitro studies examining mitochondrial respiration with neuron‐only and neuron‐astrocyte co‐cultures reveal that NMNAT2‐deficient neurons not only exhibit intrinsic mitochondrial dysfunction but also negatively influence the metabolic support provided by WT astrocytes (Figure S8). Rather than compensating for neuronal energy loss, astrocytes in co‐culture exacerbated mitochondrial impairment over time. These findings suggest that neuronal NAD deficiency induces astrocytic metabolism reprogramming, likely via altered lipid metabolism, subsequently exacerbating neuronal energetic failure. This deleterious neuron–astrocyte feedback loop may drive a self‐amplifying metabolic crisis underlying neurodegenerative progression. Future studies elucidating the molecular mediators of this bidirectional communication and the role of SARM1 in this process will be crucial for identifying therapeutic targets that restore metabolic resilience and prevent progressive neuronal loss.

An increased abundance of HK2, but not HK1, in NMNAT2 cKO cortices is a notable finding. HK2 is primarily expressed in microglia and is required for microglial glycolytic flux and energy production.^[^ ^92^, ^93^ ^]^ Since microglial activation is often accompanied by elevated glycolysis, increased HK2 expression is believed to support inflammatory responses by sustaining ATP production independently of OXPHOS.^[^ ^94^ ^]^ We observe that HK2 is upregulated in parallel to microglia activation (Figure 3 and Table S3), which is evidence of a glial response to signals from NMNAT2 KO neurons. Interestingly, HK2 antagonism slows neurodegeneration in 5XFAD mice, a commonly used AD mouse model.^[^ ^95^ ^]^ ACSS2 in the FA synthesis pathway is downregulated in NMNAT2 cKO cortices (Figure 4; Table S3). It has recently been shown that ACSS2 is predominantly expressed in oligodendrocytes,^[^ ^96^ ^]^ and its upregulation results in acetyl‐CoA increases for de novo lipogenesis and histone acetylation.^[^ ^97^ ^]^ Collectively, our data suggest that NMNAT2 loss in glutamatergic neurons induces profound metabolic reprogramming not only in neurons but also in astrocytes, microglia, and oligodendrocytes.

Impaired glycolysis or altered FAO^[^ ^33^, ^66^, ^98^ ^]^ can induce oxidative stress to activate microglia and astrocytes, subsequently triggering neuroinflammatory responses.^[^ ^99^, ^100^ ^]^ In other words, the metabolic plasticity can result in a vicious cycle by triggering chronic inflammation that worsens neuronal damage.^[^ ^101^, ^102^ ^]^ Our metabolomic analysis found elevated levels of 3‐nitrotyrosine, a marker of oxidative stress,^[^ ^103^ ^]^ in cKO cortices (normalized %, Ctrl: 100, cKO: 126.6; Difference: 26.59 ± 8.23, *p* < 0.05). Inflammatory responses with increased astrocyte reactivity and microglia numbers have been observed in many neurodegenerative conditions.^[^ ^104^, ^105^ ^]^ Future studies to understand how the disruption of neuronal NAD homeostasis triggers inflammatory responses will offer essential insights for therapeutic interventions designed to reduce inflammation‐related harm.

Astrocyte–neuron metabolic crosstalk is increasingly recognized as a critical determinant of neuronal resilience in neurodegenerative diseases.^[^ ^106^, ^107^, ^108^ ^]^ Recent transcriptomic studies by TCW et al.,^[^ ^109^ ^]^ using human brain tissue, iPSC‐derived cell models, and transgenic mice, revealed APOE4‐driven lipid metabolic deficits in astrocytes that promote glial activation and impaired astrocyte‐neuron communication. These findings highlight how disturbances in astrocytic lipid handling may propagate neuronal stress in Alzheimer's disease (AD). Multiscale proteomic network modeling further identified key protein modules and regulatory nodes associated with AD progression.^[^ ^110^ ^]^ Among these, a glia‐neuron co‐expression module encompassing proteins involved in metabolism and antigen presentation suggests tight metabolic–immune coupling across neurons, astrocytes, and microglia. Within this glia–neuron subnetwork, AHNAK, primarily expressed in astrocytes, has emerged as a potential key driver regulating signaling pathways that converge on inflammation and lipid metabolism.^[^ ^110^ ^]^ Its downregulation in APOE4/4 iPSC‐derived astrocytes exerts neuroprotective effects in co‐culture with neurons. NMNAT2, whose abundance is reduced across several neurodegenerative conditions and is proposed as an AD therapeutic target,^[^ ^111^ ^]^ may similarly sustain metabolic integrity. Together, integrating our findings with the existing literature highlights the interdependence of neuronal and astrocytic metabolism and suggest that its disruption triggers inflammatory cascades driving neurodegenerative progression.

## 4 Conclusion

Our data reveals a strong link between neuronal NAD⁺ depletion, metabolic reprogramming, and neurodegeneration. Upon NMNAT2 loss, neuronal glucose metabolism is disrupted. The brain compensates by increasing FAO and ketogenesis to meet its energy demands. However, this adaptive metabolic response disrupts normal lipid metabolism, thereby compromising neuronal function and neuron–glia communication. SARM1, localized in the mitochondria,^[^ ^112^, ^113^ ^]^ where FAO occurs, is likely to mediate this maladaptive metabolic shift accompanying NMNAT2 deficiency. Future studies using cell‐type‐specific SARM1 deletion models will be critical to delineate where and how SARM1‐mediated metabolic reprogramming occurs. Establishing causal mechanisms linking neuron–glia metabolic interactions to disease progression will be essential for developing strategies that restore metabolic balance, attenuate neuroinflammation, and prevent neurodegeneration.

## 5 Experimental Section

### Animals and Genotyping

Cortical glutamatergic neuron‐specific NMNAT2 knockout (Nex^cre/+^;NMNAT2^f/f^; cKO) mice were generated by crossing NMNAT2^f/f^ mice^[^ ^15^ ^]^ with NEX‐Cre mice (Nex^cre/+^)^[^ ^39^ ^]^ as previously described.^[^ ^13^ ^]^ Nex^cre/+^;NMNAT2^f/+^ littermates were used as the control mice (ctrl). SARM1 knockout (S^null/null^) mice^[^ ^113^ ^]^ were crossed with Nex^cre/+^;NMNAT2^f/+^ mice to generate cKO;S^null/+^ (cKO;S/+) and cKO;S^null/null^ (cKO;S/S) transgenic mice. The littermate with Nex^cre/+^;NMNAT2^f/+^ or Nex^cre/+^;NMNAT2^f/+^;S^null/+^ genotypes were used as controls (ctrl‐2) for neurodegenerative phenotyping. The littermates with Nex^cre/+^;NMNAT2^f/+^ genotype were used as control‐3 (ctrl‐3) for lipidomic analysis (no significant difference in the results from immunostaining and behavioral experiments was observed between these two genotypes). Both NMNAT2 cKO and cKO;S^null/+^ mice exhibited an ataxia phenotype, and thus, a 5g DietGel 76A (Clear H_2_O) was given daily upon weaning.

Both male and female mice were used in this study. All mice were housed under standard conditions with ad libitum access to food and water and maintained on a 12‐h light/dark cycle. Animal care and experimental procedures complied with the guidelines of the U.S. Department of Health and Human Services and were approved by the Indiana University Bloomington Institutional Animal Care and Use Committee. The genotyping procedure was conducted as previously described.^[^ ^13^ ^]^

### Tissue Preparations and Immunostaining

Mice with desired genotypes were anesthetized and transcardially perfused with 4% paraformaldehyde (PFA) in phosphate‐buffered saline (PBS). Brains were harvested and post‐fixed overnight in 4% PFA at 4 °C. After rinsing with PBS, the brains were cryopreserved in 30% sucrose in PBS at 4 °C for 1–2 days. Cryopreserved brains from P5‐P90 were mounted on the cutting platform of a Leica microtome (SM‐2000R, Leica) with dry ice and sectioned into free‐floating coronal slices at 30–40 µm thickness for subsequent staining procedures.

The immunostaining process was conducted at room temperature unless otherwise noted. Sections were washed three times for 5 min each with PBS containing 0.3% Triton (0.3% PBST), then permeabilized with 0.3% PBST for 20 min. Following permeabilization, sections were incubated in a blocking solution (3% bovine serum albumin (BSA) in 0.3% PBST) for 2 h and then incubated overnight at 4 °C with primary antibodies diluted in blocking solution. The following antibodies were used: GFAP antibody (DAKO, GA524, RRID:AB_2 811 722, Lot#: 20 071 831; 1:1000), IBA1 antibody (WAKO, 019–19741, RRID: AB_839 504, Lot#: WTP2670; 1:1000), and Neurofilament‐M antibody (Millipore, MAB1621, RRID:AB_94 294, Lot#: 2 474 859; 1:1000). The next day, sections were washed three times for 5 min each with 0.3% PBST and incubated with secondary antibodies diluted in blocking solution at 4 °C for 2 h. The following secondary antibodies were used: Alexa Fluor 488 goat anti‐Rabbit IgG (H+L) antibody (Thermo Fisher, A‐11034, RRID:AB_2 576 217, Lot#: 1 583 138, 1:1000), Alexa Fluor 488 goat anti‐Chicken IgG (H+L) antibody (Molecular Probes, A‐11039, RRID:AB_142 924, Lot#: 2 566 343, 1:1000), Alexa Fluor 488 goat anti‐Mouse IgG (H+L) antibody (Molecular Probes, A‐11029, RRID:AB_138 404, Lot#: 2 120 125, 1:1000), Alexa Fluor 555 goat anti‐Rabbit IgG (H+L) antibody (Molecular Probes, A‐21429, RRID:AB_2 535 850, Lot#: 2 790 124, 1:1000). After incubation, sections were washed three more times with 0.3% PBST. To visualize nuclei, Draq5 (Cell Signaling, 4048L, 1:10000) or DAPI (Invitrogen, D3571, 5 µg mL^−1^) was applied. Finally, sections were mounted using Dako Mounting Medium (Agilent Technologies, CS70330‐2) for imaging.

### Behavioral Assays

All behavior tests listed below were conducted under 100‐lux white‐light illumination. Mice were transferred to the testing room and acclimated for at least 1 h before tests. Experiments were performed around a similar time of day to align with the animals' biological rhythms.

### Behavioral Assays—Horizontal Ladder Test (HLT)

To assess locomotor skills, the horizontal ladder walking test was performed.^[^ ^114^ ^]^ Mice were placed on a 100 cm‐long horizontal ladder with unevenly spaced metal rungs, elevated 15 cm above the ground. The mouse's home cage was positioned at the end of the ladder to encourage movement. The camera was placed laterally to record the animal's movement along the ladder. From the recorded videos, missteps of both the left and right hind limbs were manually counted. The percentages of missing steps for both the left and right limbs relative to the total steps were calculated.

### Behavioral Assays—Rotarod Test

The rotarod test (Ugo Basile, 47 600) was conducted as previously described.^[^ ^115^ ^]^ Briefly, an individual mouse was placed on the rotating rod facing away from the direction of rotation, with an initial speed of 4 RPM. Once all mice were in place, the rotation speed was accelerated from 4 RPM to 40 RPM over 300 s. The latency to fall and the rotational speed at which the mouse fell were recorded. Each mouse underwent four consecutive trials, with a 5‐min rest period in their home cage between trials.

### Behavioral Assays—Open Field Assay

Locomotor activity was assessed using the open field assay. Mice were placed in the center of an open field arena (43 cm × 43 cm × 43 cm) enclosed by non‐transparent Plexiglas walls and allowed to move freely for 30 min. Travel distances and velocity were recorded using EthoVision XT software, with the animal's head designated as the reference point for tracking. The total distance traveled was used as a measure of general locomotor activity.^[^ ^116^ ^]^ The arena was thoroughly cleaned with 70% ethanol between trials.

### Confocal Microscopy and Quantitative Image Analysis

Confocal fluorescence images were captured with either a Leica TCS SPE confocal microscope (Leica DM 2500) using either a 10× objective lens (0.3 N.A.) or a 40× objective lens (0.75 N.A.) or a Nikon A1R laser scanning confocal microscope (Nikon A1) equipped with a 10× objective lens (0.5 N.A.) or a 60× oil‐immersion objective lens (1.4 N.A.). DAPI/Draq5 immunofluorescence was used to identify comparable anatomical regions across different brain sections. >3 sections per mouse were imaged for both hemispheres with identical imaging parameters for the antibody staining and analyzed in a consistent and comparable manner across all experimental groups. The imaging setting was chosen to minimize signal saturation across experimental groups. All quantifications were conducted blind to genotype information. DAPI or Draq5 immunofluorescence was used as a reference to draw the contours of regions of interest.

Images were analyzed using Fiji (ImageJ with updated plug‐ins). IBA1‐positive cell densities were determined by manually marking positive cells using the ImageJ Cell Counter function. For GFAP‐positive astrocyte quantification, images were thresholded using the top 3.7%–4.5% tail of total pixels, followed by “Analyze Particles” to calculate pixel percentages. Image acquisition and quantification were performed with 0.25mm^2^ area in the dorsal striatum and 0.075mm^2^ in hippocampal CA1.

For NFM‐positive axonal tracts passing through the dorsal striatum, only coronal slices from the middle rostral‐caudal axis were selected for quantification. Areas occupied by blood vessels were excluded. The vessel‐subtracted dorsal striatal regions were then processed using the “Threshold” function (top 8%–11% tail of total pixels). The “Analyze Particles” function was then applied to determine the percentage of pixels representing NFM‐positive axons.

### Brain Tissue Preparation for Metabolomic Proteomic and Lipidomic MS Analysis

All the Brain tissues were collected when mice reached postnatal day 16–21. The somatosensory cortical region was rapidly dissected immediately after euthanasia. All procedures were performed on ice to preserve tissue integrity. The dissected tissues were then snap‐frozen in a dry ice/ethanol bath and stored at −80 °C for subsequent metabolomic and proteomic mass spectrometry analysis.

### KEGG Pathway and PPI Network Analysis for Proteomic Data

Significantly up‐ or downregulated proteins (p<0.05) were used for KEGG pathway enrichment analysis through EnrichR. Irrelevant terms, such as “Malaria” and “Salmonella infection”, were removed, and the top 25 up‐ and top 15 down‐regulated pathways are shown. Similarly, significantly up‐ or downregulated proteins were used for protein–protein interaction (PPI) network construction on the STRING website, using a medium confidence (0.4) interaction score filter. The network edge thickness represents interaction confidence based on experiments, databases, and co‐expression evidence. DBSCAN clustering with a network‐specific epsilon parameter (network radius times 4) was applied. The clusters with at least three members were selected and exported to Cytoscape software for further KEGG pathway enrichment analysis and annotation. The node size represented the relative log_2_FC of each protein, while the edge width represented the interaction confidence.

### Weighted Gene Correlation Network Analysis (WGCNA) for Proteomic Data

When more than one Uniprot IDs match the same gene symbol (MGI symbol), the one with the lower unique peptide count or normalized abundance was removed. Then, two independent proteomic datasets were integrated with batch‐effect correction by the HarmonizR R package with the default ComBat method.^[^ ^117^ ^]^ Confirmed by principal component analysis, group distribution within the same batch was preserved while group difference between batches was reduced. One NMNAT2 cKO; SARM1 WT sample was excluded as an outlier, and the remaining samples’ commonly detected proteins were analyzed by the R package WGCNA.^[^ ^118^ ^]^ The unsigned Pearson correlation matrix was calculated to measure gene co‐expression similarity, and a weighted adjacency matrix was calculated by raising the correlation matrix to the power of a soft threshold equals 9. The minimum height (dissimilarity) for merging modules was set at 0.25. To define sample traits, samples with neurodegeneration were given 1, otherwise 0; samples with NMNAT2 expression were given 1, otherwise 0; SARM1 WT homozygous samples were given 2, heterozygous as 1, and KO as 0. For module‐trait correlation analysis, all samples were used for examining correlation to neurodegeneration; SARM1 WT & heterozygous samples were selected for examining correlation to NMNAT2 expression; NMNAT2 cKO samples were selected for examining correlation to SARM1 gene dosage. The Pearson correlation coefficient and correlation *p*‐value of the first principal component of the module protein expression profile (eigenprotein) and trait values were calculated to identify significant modules (*p *< 0.05) correlated to a particular trait. The genes within the module of interest were input to the STRING website for PPI visualization and pathway annotation.

### Metabolomic Mass Spectrometry Analysis

Gas chromatography–mass spectrometry (GC–MS) and liquid chromatography–mass spectrometry (LC–MS) experiments, along with metabolomic analysis, were conducted at the Metabolomics Core Facility at the University of Utah (Salt Lake City, UT, USA) (https://uofuhealth.utah.edu/diabetes‐metabolism‐research‐center/research/core‐facilities).

### GC‐MS Sample Extraction

Tissue metabolites were extracted by transferring each sample to 2.0 mL ceramic bead mill tubes (Qiagen Catalog Number 13116–50). To each sample, 450 µL of cold 90% methanol (MeOH) solution containing the internal standard d4‐succinic acid (Sigma 293 075) was added per 25 mg of tissue. Samples were homogenized using an OMNI Bead Ruptor 24 and then incubated at −20 °C for 1 h. Following incubation, samples were centrifuged at 20000 x g for 10 min at 4 °C. Subsequently, 600 µL of supernatant was transferred from each bead mill tube into labeled, fresh microcentrifuge tubes. Another internal standard, d27‐myristic acid, was added to each sample. Pooled quality control samples were generated by extracting a portion of supernatant from each sample. Process blanks consisting solely of extraction solvent underwent the same procedural steps as the actual samples. Finally, samples were dried en vacuo.

### GC‐MS

GC‐MS analyses were conducted using an Agilent 5977b GC‐MS MSD‐HES coupled with an Agilent 7693A automatic liquid sampler. Dried samples were reconstituted in 40 µL of a 40 mg mL^−1^ solution of O‐methoxylamine hydrochloride (MOX) in dry pyridine and incubated for 1 h at 37 °C. Subsequently, 25 µL of this solution was transferred to autosampler vials. An automatic addition of 60 µL of N‐methyl‐N‐trimethylsilyltrifluoracetamide (MSTFA with 1% TMCS) was performed using the autosampler, followed by incubation for 30 min at 37 °C. After incubation, samples were vortexed, and 1 µL of the prepared sample was injected into the gas chromatograph inlet in split mode with the inlet temperature maintained at 250 °C. A split ratio of 5:1 was used for most metabolites; however, metabolites saturating the instrument at this ratio were analyzed using a 50:1 split. The gas chromatograph was programmed with an initial temperature of 60 °C for 1 min, followed by a ramp of 10 °C min^−1^ to 325 °C, and held for 10 min. Separation was achieved using a 30‐meter Agilent Zorbax DB‐5MS with 10 m Duraguard capillary column, with helium as the carrier gas at a flow rate of 1 mL min^−1^.

### LC‐MS Sample Extraction

Samples were extracted in 450 µL of ice‐cold 4:1 methanol:water solution, supplemented with 0.1 µg/mL carnitine‐d9 internal standard, 1 mg/mL N‐ethylmaleimide, and 0.1% ammonium hydroxide. A process blank was prepared using 50 µL of ddH2O and 450 µL of the extraction solution, following the same extraction procedure. Homogenization of samples was carried out in a bead mill for 30 s, followed by transfer to new Eppendorf tubes and incubation at −20 °C for 1 h. Subsequently, samples were centrifuged at 20000 x g for 10 min at 4 °C, and the resulting supernatant was transferred to a fresh Eppendorf tube for further analysis.

### LC‐MS

Prior to analysis, both samples and process blanks (PBs) were transferred to PTFE autosampler vials. A pooled quality control (QC) sample was created by combining 5 µL from each individual sample. Before analysis, samples were randomized. Analysis was conducted using a SCIEX 6500 QTRAP coupled with a SCIEX Nexera UPLC system (AB SCIEX LLC, Framingham, MA, USA) operating in positive‐mode multiple reaction monitoring (MRM). Separation was achieved using a Sequant ZIC‐pHILIC 2.1×100 mm column (Millipore Sigma, Burlington, MA, USA) with a Phenomenex Krudkatcher (Phenomenex, Torrance, CA, USA). The chromatographic gradient started with an initial concentration of 99% ACN with 5% ddH2O (Buffer B) and 5% 25 mM ammonium carbonate in ddH2O (Buffer A), held for 3.3 min at a flow rate of 0.15 mL min^−1^. Buffer B was then decreased to 15% over 7.5 min and held for 3.8 min. Finally, Buffer B was returned to its initial concentration over 0.1 min, and the system was allowed to re‐equilibrate for 15 min between runs.

### MS Data Analysis

The GC‐MS data were collected using MassHunter software (Agilent), while metabolites were identified and their peak areas recorded using MassHunter Quant. LC‐MS data were collected and analyzed using SCIEX MultiQuant software. Metabolite identities were determined using transitions from Metlin (https://metlin.scripps.edu/landing_page.php?pgcontent = mainPage) and retention time data from in‐house pure standards. Subsequently, the data were transferred to a Microsoft Excel spreadsheet. Metabolite identity was confirmed using a combination of an in‐house metabolite library developed with pure purchased standards, the NIST library, and the Fiehn library. Statistical analysis and pathway mapping were performed using MetaboAnalyst (https://www.metaboanalyst.ca/).^[^ ^119^ ^]^

### Proteomic Mass Spectrometry Analysis

The proteomic mass spectrometry analysis conducted in this study was carried out by the Indiana University School of Medicine Proteomics Core (Indianapolis, IN, USA) (https://medicine.iu.edu/service‐cores/facilities/proteomics).

### Proteomic Sample Preparation

The flash‐frozen tissue samples were crushed using a CryoPrep homogenizer (Covaris). Subsequently, the samples were centrifuged at 14000 x g for 20 min, and protein concentrations were determined using the Bradford protein assay (BioRad Cat No: 5 000 006). Each sample containing 50 µg equivalent of protein underwent treatment with 5 mM tris(2‐carboxyethyl) phosphine hydrochloride (Sigma–Aldrich Cat No: C4706) to reduce disulfide bonds, followed by alkylation of the resulting free cysteine thiols with 10 mM chloroacetamide (Sigma–Aldrich Cat No: C0267). The samples were then diluted with 50 mM Tris.HCl pH 8.5 (Sigma‐Aldrich Cat No: 10 812 846 001) to a final urea concentration of 2 M for overnight Trypsin/Lys‐C digestion at 35 °C, using a 1:50 protease to substrate ratio (Mass Spectrometry grade, Promega Corporation, Cat No: V5072)

### Peptide Purification and Labeling

The digested samples were acidified with trifluoroacetic acid (TFA, 0.5% v/v) and subjected to desalting using Waters Sep‐Pak Vac cartridges (Waters Cat No: WAT054955), with a 1 mL wash of 0.1% TFA followed by elution in 0.6 mL of 70% acetonitrile with 0.1% formic acid (FA). The resulting peptides were dried using a speed vacuum and resuspended in 50 mM triethylammonium bicarbonate. Subsequently, peptide concentrations were assessed using the Pierce Quantitative colorimetric assay (Cat No: 23 275). Equal amounts of peptide from each sample were labeled with 0.5 mg of Tandem Mass Tag Pro (TMTpro) reagent (16‐plex kit, Thermo Fisher Scientific, TMTpro Isobaric Label Reagent Set; Cat No: 44 520, lot no. YG370070 and YI372118, according to the manufacturer's instructions (Li et al., 2020). After confirming >98% labeling efficiency, reactions were quenched with 0.3% hydroxylamine (v/v) at room temperature for 15 min. The labeled peptides were then combined, dried using a speed vacuum, and desalted to remove excess label using a 100 mg Waters SepPak cartridge, eluted in 70% acetonitrile with 0.1% formic acid, and lyophilized to dryness.

### High pH Basic Fractionation

The combined samples were resuspended in 10 mM ammonium formate at pH 10. Half of each mixture underwent fractionation using an offline Thermo UltiMate 3000 HPLC system equipped with a Waters Xbridge C18 column (3.5 µm x 4.6 mm x 250 mm, Cat No: 186 003 943). The elution gradient consisted of Buffer A (10 mM formate at pH 10) and Buffer B (95% acetonitrile with 10 mM formate at pH 10) at a flow rate of 1 mL/min: 0–15% B over 5 min, 15%–20% B over 5 min, 20%–35% B over 75 min, 35%–50% B over 5 min, and 50%–60% B over 10 min, followed by a 6‐min hold at 60% B. Fractions were collected continuously every 60 s into 96‐well plates. Initial and late fractions with minimal material were combined and lyophilized, while the remaining fractions were concatenated into 24 fractions, dried, and resuspended in 50 µL of 0.1% formic acid prior to online LC‐MS analysis.

### Nano‐LC‐MS/MS

Mass spectrometry analysis was conducted using an EASY‐nLC 1200 HPLC system (SCR: 01 4993, Thermo Fisher Scientific) coupled with an Exploris 480 mass spectrometer featuring a FAIMSpro interface (Thermo Fisher Scientific). One‐fifth of each fraction was injected onto a 25 cm Aurora column (Ion Opticks) at a flow rate of 350 nL/min. The chromatographic gradient was composed of mobile phases A (0.1% formic acid (FA) in water) and B (0.1% FA, 80% acetonitrile (Thermo Fisher Scientific Cat No: LS122500)). The gradient increased from 8% to 38% B over 98 min, followed by a 10‐min ramp to 80% B, held for 2 min, and then decreased from 80% to 4% B over the final 5 min. Mass spectrometry was conducted in positive ion mode with a default charge state of 2, employing advanced peak determination and a lock mass of 445.12003. Three FAIMS CVs (‐45, ‐55, and ‐65 CV) were utilized, each with a cycle time of 1 s and identical MS and MS2 parameters. Precursor scans (*m/z* 375–1500) were performed at an orbitrap resolution of 120 000, with an RF lens% of 40, automatic maximum injection time, standard AGC target, and a minimum MS2 intensity threshold of 5e3. Dynamic exclusion was set at 30 s. MS2 scans were conducted with a quadrupole isolation window of 1.6 *m/z*, 30% HCD collision energy, a resolution of 15 000, standard AGC target, automatic maximum injection time, and a fixed first mass of 110 *m/z*.

### Proteomic Mass Spectrometry Data Analysis

Resulting RAW files were analyzed in Proteome Discover 2.5 (Thermo Fisher Scientific) with a Mus musculus UniProt reference proteome FASTA (downloaded 02 2823, 55 250 sequences) plus common laboratory contaminants (73). SEQUEST HT searches were conducted with full trypsin digest, 3 maximum number missed cleavages; precursor mass tolerance of 10 ppm; and a fragment mass tolerance of 0.02 Da. Static modifications used for the search were, 1) carbamidomethylation on cysteine (C) residues; 2) TMTpro label on lysine (K) residues. Dynamic modifications used for the search were TMTpro label on N‐termini of peptides, oxidation of methionines, deamidation of asparagine or arginine, phosphorylation on serine, threonine, or tyrosine, and acetylation, methionine loss, or acetylation with methionine loss on protein N‐termini. Percolator False Discovery Rate was set to a strict setting of 0.01 and a relaxed setting of 0.05. IMP‐ptm‐RS node was used for all modification site localization scores. Values from both unique and razor peptides were used for quantification. In the consensus workflows, peptides were normalized by total peptide amount with no scaling. Quantification methods utilized TMTpro isotopic impurity levels available from Thermo Fisher Scientific. Reporter ion quantification was allowed with an S/N threshold of 6 and a co‐isolation threshold of 30%. Resulting grouped abundance values for each sample type, abundance ratio values, and respective *p*‐values (*t*‐test) from Proteome Discover were exported to Microsoft Excel.

### Lipidomic Mass Spectrometry Analysis

The lipidomic mass spectrometry analysis conducted in this study was carried out by Metware Biotechnology Inc. (Woburn, MA, USA) (www.metwarebio.com). Ultra‐performance liquid chromatography‐tandem mass spectrometry (UPLC‐MS/MS) was performed for qualitative and quantitative compound analysis.

### Lipidomic Sample Preparation

Take out the sample from the −80 °C refrigerator and thaw it on ice. Multi‐point sample and weigh 20 mg of sample, homogenize (30 HZ) for 20 s with a steel ball and the centrifuge (3000 rpm, 4 °C) for 30 s. Then, add 1 mL of the extraction solvent (MTBE: MeOH = 3:1, v/v) containing the internal standard mixture. After whirling the mixture for 15 min, 200 µL of ultrapure water was added. Vortex for 1 min and centrifuge at 12 000 rpm for 10 min. 200 µL of the upper organic layer was collected and evaporated using a vacuum concentrator. The dry extract was dissolved in 200 µL reconstituted solution (ACN: IPA = 1:1, v/v) to LC‐MS/MS analysis.

### Chromatography‐Mass Spectrometry Acquisition Conditions

The data acquisition instruments consisted of Ultra Performance Liquid Chromatography (UPLC) (Nexera LC‐40, https://www.shimadzu.com) and tandem mass spectrometry (MS/MS) (Triple Quad 6500+, https://sciex.com/).

### Chromatography‐Mass Spectrometry Acquisition Conditions—Liquid Phase Conditions

1. Chromatographic column: Thermo AccucoreC30 (2.6 µm, 2.1 mm×100 mm i.d.)
2. Mobile phase: A phase was acetonitrile /water (60/40, V/V) (0.1% formic acid added, 10 mmol L^−1^ ammonium formate); B phase was acetonitrile / Isopropyl alcohol (10/90, V/V) (0.1% formic acid added, 10 mmol L^−1^ ammonium formate)
3. Gradient program: 80:20(V/V) at 0 min, 70:30(V/V) at 2 min, 40:60(V/V) at 4 min, 15:85(V/V) at 9 min, 10:90(V/V) at 14 min, 5:95(V/V) at 15.5 min, 5:95(V/V) at 17.3 min, 80:20(V/V) at 17.5 min, 80:20(V/V) at 20 min;
4. Flow rate: 0.35 mL min^−1^; Column temperature: 45 °C; Injection volume: 2 µL.

### Chromatography‐Mass Spectrometry Acquisition Conditions—Mass Spectrometry Conditions

LIT and triple quadrupole (QQQ) scans were acquired on a triple quadrupole‐linear ion trap mass spectrometer (QTRAP), QTRAP 6500+ LC‐MS/MS System, equipped with an ESI Turbo Ion‐Spray interface, operating in positive and negative ion mode and controlled by Analyst 1.6.3 software (Sciex). The ESI source operation parameters were as follows: ion source, turbo spray; source temperature 500 °C; ion spray voltage (IS) 5500 V(Positive), ‐4500 V (Neagtive); Ion source gas 1 (GS1), gas 2 (GS2), curtain gas (CUR) were set at 45, 55, and 35 psi, respectively. Instrument tuning and mass calibration were performed with 10 and 100 µmol/L polypropylene glycol solutions in QQQ and LIT modes, respectively. QQQ scans were acquired as MRM experiments with collision gas (nitrogen) set to 5 psi. DP and CE for individual MRM transitions were done with further DP and CE optimization. A specific set of MRM transitions was monitored for each period according to the lipids eluted within this period.

### Principles of Lipid Qualification and Quantification

With the in‐house database MWDB (www.metwarebio.com), lipids were annotated based on their retention time and ion‐pair information from MRM mode. In MRM mode, the first quadrupole screens the precursor ions for the target substance and excludes ions of other molecular weights. After ionization induced by the impact chamber, the precursor ions were fragmented, and a characteristic fragment ion was selected through the third quadrupole to exclude the interference of other non‐target ions. By selecting a particular fragment, quantification is more accurate and reproducible.

### Quantitative Real‐Time PCR

Cortical, olfactory bulb, and cerebellar tissues were collected and immediately snap‐frozen at −80 °C until use. Samples included four male and one female mouse from each genotype (control and NMNAT2 cKO). Total RNA was extracted using the RNeasy Plus Universal Kit (Qiagen, 73 404) with on‐column DNase digestion according to the manufacturer's instructions. RNA concentration and purity were assessed using a NanoDrop 2000 spectrophotometer (Thermo Fisher Scientific). For cDNA synthesis, 500 ng of total RNA was reverse transcribed using the TaqMan Reverse Transcription Reagents kit (Invitrogen, N8080234).

Quantitative PCR was performed using the QuantStudio 7 Flex Real‐Time PCR System (Thermo Fisher Scientific). For NMNAT2 detection, reactions contained 450 nM forward primer, 450 nM reverse primer, and 150 nM probe (PrimeTime qPCR Probe Assay, Integrated DNA Technologies, Assay ID: Mm.PT.58.11222642). GAPDH was used as an internal control (PrimeTime Predesigned qPCR Assay, IDT, Assay ID: Mm.PT.39a.1) with identical reporter and quencher chemistry. Each 10 µL reaction contained 0.3 µL cDNA, 500 nM forward and reverse primers, and 250 nM probe.

Cycle threshold (Ct) values were determined automatically. ΔCt was calculated as the difference between NMNAT2 and GAPDH Ct values, and ΔΔCt was obtained by normalizing to the mean ΔCt of control samples. Relative expression was calculated using the 2^−ΔΔCt^ method.

### Astrocyte Isolation by MACs Sorting

Cortices from P16 mice of the indicated genotypes were rapidly dissected and enzymatically dissociated using the Adult Brain Dissociation Kit (Miltenyi Biotec, 130‐107‐677) and the gentleMACS Dissociator (Miltenyi Biotec, 130‐093‐235), following the manufacturer's protocol. Cell suspensions were passed through 70‐µm strainers, followed by debris removal (Miltenyi Biotec, 130‐109‐398) and dead cell removal (Miltenyi Biotec, 130‐090‐101) procedures. Cells were then pelleted and resuspended in D‐PBS^−/−^ buffer containing 0.5% BSA. Astrocytes were isolated using magnetic‐activated cell sorting (MACS) with anti‐ACSA‐2 MicroBeads (Miltenyi Biotec, 130‐097‐678) according to the manufacturer's instructions. The purity of sorted astrocytes was confirmed by qPCR for cell‐type‐specific markers (e.g., Aldh1l1, Gfap, Tmem119, Rbfox3).

### RNA‐Seq Sample Preparation and Analysis

Total RNA was extracted from freshly isolated astrocytes using the RNeasy Mini Kit (Qiagen, 74 104) with on‐column DNase digestion. RNA concentration, purity, and integrity were assessed using a NanoDrop 2000 spectrophotometer (Thermo Fisher Scientific); all samples had RNA integrity numbers (RIN) ≥8.0. Purified RNA samples were submitted to the Center for Medical Genomics at Indiana University School of Medicine (Indianapolis, IN, USA; https://medicine.iu.edu/service‐cores/facilities/medical‐genomics) for library preparation, sequencing, and bioinformatic analysis.

### Astrocyte Culture

Cryopreserved primary mouse astrocytes (ScienCell, Cat. # M1800) were thawed quickly in a 37 °C water bath and plated immediately onto poly‐L‐lysine–coated vessels (2 µg cm^−2^). Cells were cultured in Astrocyte Medium (ScienCell) under standard conditions (37 °C, 5% CO_2_). The medium was refreshed the next day to remove residual DMSO and unattached cells, then changed every 3 days until ∼70% confluence, and thereafter every other day until ∼90% confluence. Subculturing was performed when cultures reached 90%–95% confluence using 0.05% trypsin/EDTA (ScienCell), with gentle handling to avoid over‐digestion. Astrocytes were used at early passages (within 5 population doublings) for co‐culture, as described in Seahorse CF Cell Mito Stress Test.

### Primary Neuronal Culture

Primary cortical neurons were prepared from E15.5–E16.5 embryos obtained from NMNAT2‐Blad heterozygous intercrosses, as previously described.^[^ ^13^ ^]^ Embryos of both sexes were used for culture preparation. Cortices were dissected and individually dissociated using the Worthington Papain Dissociation System (Worthington Biochemical Corporation) according to the manufacturer's instructions. Genotyping was performed prior to the completion of papain digestion. Cortical tissues from multiple NMNAT2‐Blad wild‐type (WT) embryos were pooled as the WT group, while cortices from homozygous embryos were pooled as the knockout (KO) group.

Following dissociation, neurons were plated onto poly‐D‐lysine‐coated culture plates or coverslips and maintained in Neurobasal Medium (Gibco, Thermo Fisher Scientific) supplemented with 2% B‐27 (Gibco), 2 mM GlutaMAX (Gibco), and 100 U/mL penicillin–streptomycin (Gibco). Cultures were incubated at 37 °C in a humidified 5% CO_2_ atmosphere. One‐third of the medium was replaced every 3 days.

### Seahorse XF Cell Mito Stress Test Assay

Mitochondrial function was assessed using the Seahorse XF Cell Mito Stress Test Kit (Agilent, 103015–100) according to the manufacturer's protocol. Primary cortical neurons and astrocytes were prepared as described above. Neurons were seeded in Seahorse XF24 Cell Culture Microplates at a density of 40 000 cells per well. Astrocytes were added at DIV2 at a 1:10 astrocyte‐to‐neuron ratio (4000 astrocytes per well). Assays were performed at DIV8, DIV10, and DIV12 using the Seahorse XF Flex Analyzer (Agilent Technologies).

Sequential injections of mitochondrial modulators were performed at the following final concentrations: 1.5 µM oligomycin, 2 µM FCCP, and 0.5 µM rotenone/antimycin A. Oxygen consumption rate (OCR) was recorded and analyzed using Wave software (Agilent Technologies) following the manufacturer's instructions. Data were normalized to total protein content per well as determined by bicinchoninic acid (BCA) assay.

### Statistical Analysis

All data are presented as mean ± SEM. Sample sizes (*n* = 3–12 per group, as indicated) were determined based on the exploratory scope of the study and practical feasibility. Exact sample sizes for each experiment are provided in the figure legends and in the Supplementary Statistics Table. Statistical analyses were performed using GraphPad Prism (v10.0.0; GraphPad Software, Boston, MA, USA). Differences between the two groups were assessed using two‐tailed unpaired Student's *t*‐tests. For comparisons involving more than two groups, one‐way or two‐way ANOVA was applied. When data did not meet the assumptions of normality or equal variance, nonparametric tests (Mann–Whitney or Kruskal–Wallis) were used instead of t‐tests or ANOVA, respectively. A p‐value < 0.05 was considered statistically significant. Significance levels are indicated as follows: *, *p* < 0.05; **, *p* < 0.01; ***, *p* < 0.001; ****, *p* < 0.0001.

## Conflict of Interest

The authors declare no conflict of interest.

## Author Contributions

H.C.L., Z.X.N., and J.Y.H. designed the study, Z.X.N., A.E., and N.E. performed the experiments, Z.X.N., S.Y., A.E., N.E., and C.H. analyzed data. H.C.L., A.E., N.E., S.Y., and Z.X.N. wrote the manuscript with inputs from C.S.W., J.M.T., and J.Y.H.

## Supporting information



Supporting Information

Supplemental Table 1

Supplemental Table 2

Supplemental Table 3

Supplemental Table 4

Supplemental Table 5

Supplemental Figures

## Data Availability

The data that support the findings of this study are available from the corresponding author upon reasonable request. The astrocyte RNA‐seq data generated in this study have been deposited in the Gene Expression Omnibus (GEO) under accession GSE312242. The cortical proteomic dataset is publicly available through the ProteomeXchange Consortium via the MassIVE repository with accession MSV000099730 and is cross‐referenced as PXD070236. The cortical lipidomic datasets are currently being deposited into Metabolomics Workbench. The accession number will be provided once the submission is finalized.
