# Supplementary material for: NAD⁺ Reduction in Glutamatergic Neurons Induces Lipid Catabolism and Neuroinflammation in the Brain via SARM1
Source: Adv Sci (Weinh). 2025 Dec 12;13(6):e09950. doi: 10.1002/advs.202509950 (PMC12866834; doi:10.1002/advs.202509950)
Supplement: Supplementary file 1 — Supporting Information [file ADVS-13-e09950-s003.docx]

**Supplementary Figures**

**Figure S1**

Altered ketogenesis and ketolysis in NMNAT2 cKO cortices. **(A)** Scheme of ketogenesis and ketolysis pathway. Metabolites labeled with a dashed line were not detected in the MS analysis. **(B)** Heatmaps of log2-transformed values summarize the enzymes of ketone metabolism (n=4 males per group). **(C)** Summary of normalized percentage of 3-Hydroxybutyric acid (BHB). Sample size: P16-P18 ctrl and cKO, n = 6 per sex. *, p<0.05 by Student’s t-test. *See Supplementary Statistics Table for detailed statistics.* Abbreviations: ACAT1, acetyl-CoA acetyltransferase; BDH1, D-beta-hydroxybutyrate dehydrogenase; HMGCL, hydroxymethylglutaryl-CoA lyase; HMGCS2, hydroxymethylglutaryl-CoA synthase; OXCT1, succinyl-CoA:3-ketoacid coenzyme A transferase 1.

**Figure S2**

Summary of lipidomic analyses. **(A,C)** Principal component analysis (PCA) reveals distinct separation of lipidomic profiles among genotypes. **(B,D)** Volcano plots show differentially expressed lipids upon NMNAT2 loss and the extent of recovery following SARM1 deletion.

**Figure S3**

Neuronal NMNAT2 loss increases astrocyte reactivities upon. **(A)** Representative GFAP staining images from P21 ctrl and NMNAT2 cKO hippocampus and striatum. **(B)** Bar graphs summarize normalized % of GFAP+ area at P4/5 (ctrl, n=3; cKO, n=4), P16/21 (n=6 per group), and P90 (n=3 per group) **(C)** High-magnification images with enlarged views (inserts) showing morphology of GFAP positive astrocytes in P21 corpus callosum, hippocampus, striatum, and cortex of ctrl and cKO brains. Almost no GFAP signals are present in upper cortical regions. Draq5 signals reveal nuclei locations. *, p<0.05; **, p<0.01; ***, p<0.001; ****, p<0.0001 by Student’s t-test. *See Supplementary Statistics Table for detailed statistics.*

**Figure S4**

Neuronal NMNAT2 loss increases the number of microglia and reactivities. **(A)** Representative IBA1 staining images from the hippocampus and striatum of NMNAT2 cKO and littermate control mice. **(B)** Bar graphs summarize the percentage of IBA1+ densities to controls at P4/5 (ctrl, n=3; cKO, n=4), P16/21 (n=6 per group), and P90 (n=3 per group). **(C)** High magnification images with enlarged views (inserts) showing IBA1+ microglia morphology in P21 corpus callosum, hippocampus, striatum, and cortex of control and cKO brains. *, p<0.05; **, p<0.01; ***, p<0.001; ****, p<0.0001 by Student’s t-test.

**Figure S5**

Complete SARM1 deletion ameliorates neuroinflammatory responses in NMNAT2 cKO brains. (**A**) Representative GFAP staining images in the hippocampus and striatum of the three genotypes. (**B**) Summary of normalized % of GFAP+ densities in hippocampus and striatum (ctrl-2, n=10; cKO;S/+, n=9; cKO;S/S, n=5). (**C**) Representative IBA1 staining images from the hippocampus and striatum at P21. (**D**) Summary of normalized % of IBA1+ cell densities in hippocampus and striatum (ctrl-2, n=10; cKO;S^null/+^, n=9; cKO;S^null/null^, n=5) *, p<0.05; **, p<0.01; ***, p<0.001; ****, p<0.0001 by Kruskal-Wallis test. Abbreviations: ctrl-2, control-2; cKO;S/+, cKO;S^null/+^; cKO;S/S, cKO;S^null/null^. *See Supplementary Statistics Table for detailed statistics.*

**Figure S6**

Weighted gene co-expression network analysis (WGCNA) of the proteomic dataset identifies modules correlated with neurodegeneration and NMNAT2 abundance. **(A)** The analysis identified 36 co-expressed protein modules. Gene dendrogram showing hierarchical clustering of proteins and corresponding module color assignments. **(B)** Module–trait correlation heatmap depicting Pearson correlation coefficients between each module and individual traits, including neurodegeneration, NMNAT2 allele, and SARM1 allele. The top modules are shown in the main Figure 8A; the remaining modules are presented here for completeness. Color intensity indicates correlation strength. *, p < 0.05; **, p < 0.01; ***, p < 0.001. **(C)** Examples of individual protein expression patterns across samples for highlighted modules. Absolute log₁₀-transformed fold changes were derived from the original protein abundance values prior to batch-effect correction.

**Figure S7**

Transcriptomic profiling of astrocytes of P16 ctrl, cKO;S/+ and cKO;S/S brains. Astrocytes were isolated using magnetic-activated cell sorting (MACS) method. **(A)** Clustering analysis for all samples used for RNAseq. **(B-D)** Smear plots showing the differential expressions between cKO;S/+ to ctrl **(B)**, (cKO;S/+ to cKO;S/S **(C)**, and cKO;S/S to ctrl **(D)**. The x-axis represents log fold change (logFC), and the y-axis represents average log counts per million (logCPM); red dots indicate significantly differentially expressed genes (FDR < 0.05). **(E)** Venn diagram illustrates the overlap of differential expressed genes among three different comparisons. **(F)** Expression levels of neuronal, astroglial, and microglial marker genes within this dataset. Only astrocyte marker genes show elevated expression, confirming successful enrichment of astrocytes.

**Figure S8**

Seahorse XF analysis of mitochondrial respirations in wild-type (WT) and NMNAT2 KO neurons with or without the presence of WT astrocytes. Four types of primary cultures were prepared to examine mitochondrial function: WT neurons, KO neurons, WT neurons mixed with WT astrocytes, and KO neurons mixed with wild-type astrocytes. Oxygen consumption rate (OCR) measurements were obtained at day-in-vitro (DIV) 8, 10, and 12 using the Agilent Seahorse Mito Stress Test. **(A-C)** Summary graphs plot for basal **(A)**, ATP-linked **(B)**, and maximal **(C)** respiration rates measured from 4 different cultures at DIV8, DIV10, and DIV 12. n = 10 values for each culture per DIV came from individual culture wells of 2 independent experiments. Student’s t-test is used for statistical comparisons. **(D–E)** Normalized % of OCR with time plots show % of changes upon the sequential injections of oligomycin (O), FCCP (F), and rotenone/antimycin A (R/A) for cultures at DIV8 (**D**) and DIV12 (**E).** The time of injections is indicated by a vertical dashed line. OCRs were normalized to the basal values of individual cultures to show % of changes at different states. Two-way repeated-measures ANOVA). Data are presented as mean ± SEM. *, p < 0.05; **, p < 0.01; ***, p < 0.001; ****, p < 0.0001. *, indicates comparisons between WT and KO neurons; #, indicates significant differences between neuron-only and neuron + astrocyte co-cultures of the same neuronal genotype at the same DIV. *See Supplementary Statistics Table for detailed statistics.*

**Supplementary tables**

**Table S1**.

The list of fold changes of individual lipid subclasses for cKO to control and cKO;S^null/+^ to cKO;S^null/null^ cortices. Arrows indicate the direction of regulation (upregulation or downregulation). *, p<0.05; **, p<0.01; ***, p<0.001; ****, p<0.0001 by Student’s t-test. 4 male mice per genotype per group.

**Table S2**.

The top 15 KEGG pathways enrichment analysis with significantly altered lipids. Pathways are ranked by the number of significant lipid compounds detected. 4 male mice per genotype per group.

**Table S3**.

Summary for proteomic changes on selected proteins involved in lipid metabolism, inflammation responses, and glutathione metabolism. Arrows indicate the direction of regulation. *, p<0.05; **, p<0.01; ***, p<0.001 by Student’s t-test.

**Table S4.**

Lists of biological processes and proteins in Plum2 and Steelblue modules

Summary of MGCNA protein analysis and selected module characteristics. N= 4 male mice per genotype per group. cKOA refers to cKO;S^null/+^ mice, and cKOB refers to cKO;S^null/Snull^ mice.

**Table S5.**

Statistics and numbers for figures and supplementary figures.
